# Drug-coated balloon: an effective alternative to stent strategy in small-vessel coronary artery disease—a meta-analysis

**DOI:** 10.3389/fcvm.2023.1213992

**Published:** 2023-08-21

**Authors:** Dominik Felbel, Filip Bozic, Benjamin Mayer, Marvin Krohn-Grimberghe, Michael Paukovitsch, Sascha d’Almeida, Johannes Mörike, Birgid Gonska, Armin Imhof, Dominik Buckert, Wolfgang Rottbauer, Sinisa Markovic, Tilman Stephan

**Affiliations:** ^1^ Department of Cardiology, Angiology, Pneumology and Internal Intensive Care, University of Ulm, Ulm, Germany; ^2^ Institute for Epidemiology and Medical Biometry, Ulm University, Ulm, Germany

**Keywords:** drug-eluting stent, small-vessel coronary artery disease, drug-eluting balloon, drug-eluting stents (DES), drug-eluting stent–drug-coated balloon

## Abstract

**Background:**

Small-vessel coronary artery disease (CAD) is frequently observed in coronary angiography and linked to a higher risk of lesion failure and restenosis. Currently, treatment of small vessels is not standardized while having drug-eluting stents (DES) or drug-coated balloons (DCBs) as possible strategies. We aimed to conduct a meta-analytic approach to assess the effectiveness of treatment strategies and outcomes for small-vessel CAD.

**Methods:**

Comprehensive literature search was conducted using PubMed, Embase, MEDLINE, and Cochrane Library databases to identify studies reporting treatment strategies of small-vessel CAD with a reference diameter of ≤3.0 mm. Target lesion revascularization (TLR), target lesion thrombosis, all-cause death, myocardial infarction (MI), and major adverse cardiac events (MACE) were defined as clinical outcomes. Outcomes from single-arm and randomized studies based on measures by means of their corresponding 95% confidence intervals (CI) were compared using a meta-analytic approach. Statistical significance was assumed if CIs did not overlap.

**Results:**

Thirty-seven eligible studies with a total of 31,835 patients with small-vessel CAD were included in the present analysis. Among those, 28,147 patients were treated with DES (24 studies) and 3,299 patients with DCB (18 studies). Common baseline characteristics were equally distributed in the different studies. TLR rate was 4% in both treatment strategies [0.04; 95% CI 0.03–0.05 (DES) vs. 0.03–0.07 (DCB)]. MI occurred in 3% of patients receiving DES and in 2% treated with DCB [0.03 (0.02–0.04) vs. 0.02 (0.01–0.03)]. All-cause mortality was 3% in the DES group [0.03 (0.02–0.05)] compared with 1% in the DCB group [0.01 (0.00–0.03)]. Approximately 9% of patients with DES developed MACE vs. 4% of patients with DCB [0.09 (0.07–0.10) vs. 0.04 (0.02–0.08)]. Meta-regression analysis did not show a significant impact of reference vessel diameter on outcomes.

**Conclusion:**

This large meta-analytic approach demonstrates similar clinical and angiographic results between treatment strategies with DES and DCB in small-vessel CAD. Therefore, DES may be waived in small coronary arteries when PCI is performed with DCB.

## Introduction

Small-vessel coronary artery disease (CAD) is frequently observed in coronary angiography and has been documented in 30%–50% of cases, depending on its definition and the studied patient population (1, 2). Despite the limited extent of ischemia, revascularization is often required in symptomatic patients or after evidence of relevant myocardial ischemia (3–5). Notwithstanding many advances in interventional cardiology, small-vessel disease (SVD) still remains a challenging lesion subset to treat (6). Compared with larger coronary arteries, percutaneous coronary intervention (PCI) in small caliber vessels was associated with an increased risk of adverse clinical and angiographic events, especially with higher restenosis rates, late lumen loss, and consecutive revascularization procedures (6–9). Although previous studies evaluating newer-generation drug-eluting stents (DES) and lately drug-coated balloons (DCBs) have shown auspicious results, no standardized guideline recommendation for the optimal treatment strategy of SVD is recorded (10). DCBs are primarily applied in the treatment of in-stent restenosis (ISR) (11) and allow fast and high-dose delivery of antiproliferative drugs without using intravascular foreign material resulting in a reduced duration of dual antiplatelet therapy. These circumstances and positive vascular remodeling emphasize advantages when using DCBs compared with DES use (12–16). However, in patients with SVD, the clinical effectiveness and long-term outcome following DCB application are still a matter of debate due to inconsistent results of randomized trials comparing the two treatment approaches, ultimately leading to uncertainty as to which strategy is best (17, 18).

Therefore, we conducted a meta-analytic approach to comprehensively evaluate available treatment strategies and outcomes in SVD, especially to compare the effectiveness and safety of DCB with DES.

## Methods

### Data sources and study selection

**Figure 1:**
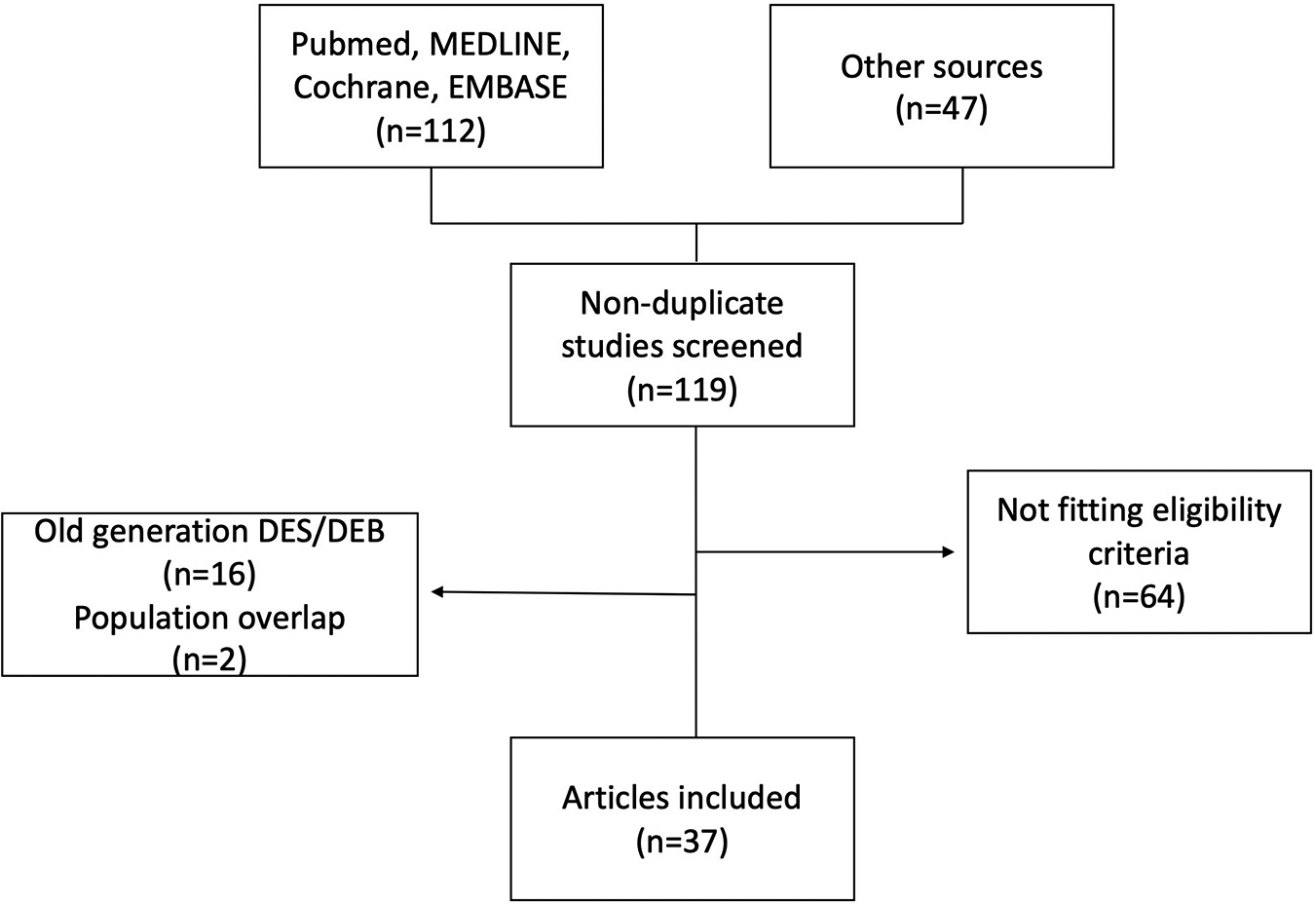
Flow diagram of study selection.

A systematic and comprehensive literature search was conducted for studies reporting treatment strategies and outcomes of small-vessel coronary artery disease using PubMed, Embase, MEDLINE, and Cochrane Library databases up to April 2020. The following terms and keywords were used in various combinations: small-vessel coronary artery disease, small-vessel disease, small coronary artery disease, small coronary vessel, drug-coated balloon, drug-eluting balloon, drug-coated stent, and drug-eluting stent. In addition, previous related meta-analyses and reviews and all references of selected articles were screened to identify any relevant studies. No sample size restriction was enforced. Figure 1 displays the literature search flow chart.

We included both randomized controlled trials (RCTs) and single-arm studies investigating treatment strategies and outcomes of small-vessel coronary artery disease with a reference diameter of ≤3.0 mm. Trials investigating PCI using DES or drug-eluting balloon (DEB) were included, because so far DES are the recommended strategy for the treatment of native coronary stenosis and DCB has evolved as a potential alternative in in-stent restenosis and lesions located in small coronary vessels.

We excluded studies on the basis of the following criteria: studies without reliable data, overlapping data, case reports, conference abstracts, review articles, and only abstract available.

### Data extraction and study quality

Two investigators independently reviewed all search results separately and selected the studies in accordance to inclusion and exclusion criteria. When a consensus was not reached between the two authors, a third reviewer was consulted for final decision.

For each eligible trial, we extracted data including article information (first author, year of publication), study characteristics (study design, arms and treatment regimes, number of patients, follow-up time; see more in Table 1), relevant population demographics [diabetes, hypertension, dyslipidemia, gender, smoking, previous myocardial infarction (MI), previous PCI, coronary artery bypass graft (CABG), and age], lesion characteristics, bail-out stenting, and interventions as well as clinical outcomes of interest. The study quality was assessed using the National Institutes of Health Quality Assessment Tool, and studies were rated as “good,” “fair,” or “poor” quality (see more in Supplementary Table S1) (52).

**Table 1:** Study characteristics.

Study	Study type	DES type or POBA	DCB type	Bail-out stenting following DEB	Sample size (balloon/DES or POBA)	Reference vessel, mm	Reference vessel diameter mean ± SD or median (IQR), mm (DCB/DES)	Clinical follow-up time
PICCOLETO	RCT	Paclitaxel-eluting stent (Taxus)	Paclitaxel-coated balloon	10 (34.5%)	28/29	≤2.75	2.45 ± 0.28	9 months
Cortese et al. (17)							2.36 ± 0.25	
BELLO	RCT	Paclitaxel-eluting stent (Taxus Libertè)	Paclitaxel-coated balloon (IN.PACT Falcon)	19 (20.2%)	90/92	<2.8	2.41 ± 0.34	6 months
Latib et al. (18, 19)							2.41 ± 0.40	
Giannini et al. (20)	Retrospective PSM	Everolimus-eluting stent (XIENCE V, Abbott) or (Promus, Boston Scientific)	Paclitaxel-coated balloon (IN.PACT Falcon)	19 (20.2%)	90/91	<2.8	2.49 ± 0.2	12 months
							2.5 ± 0.2	
Sim et al. (21)	Retrospective	Everolimus-eluting stent (XIENCE Xpedition SV or XIENCE Alpine (Abbott Vascular) and zotarolimus-eluting (Resolute Onyx, Medtronic)	Paclitaxel-coated balloons [SeQuent Please and SeQuent Please Neo (Braun) and IN-PACT Falcon (Medtronic)]	7 (8.1%)	87/200	≤2.00	1.88 ± 0.38	12 months
							1.95 ± 0.21	
Sinaga et al. (22)	Retrospective	Zotarolimus-eluting (Resolute Integrity, Medtronic), everolimus-eluting (XIENCE, Abbott), Promus Element, Boston Scientific), and Biolimus (BioMatrix, Biosensors)	Paclitaxel-coated balloon (SeQuent Please, Braun)	na	172/163	≤2.5	2.22 ± 0.30	12 months
							2.44 ± 0.19	
BASKET-SMALL 2	RCT	Everolimus-eluting XIENCE (Abbott) or placlitaxel-eluting Taxus Element (Boston)	Paclitaxel-coated balloon SeQuent Please (Braun)	na	382/376	<3.0	2.57 ± 0.25	12 months
Jeger et al. (23)							2.75 ± 2.14	
RESTORE SVD China	RCT	RESOLUTE Integrity DES (Medtronic)	Restore DCB (Cardionovum)	6 (5.2%)	115/109	≥2.25 and ≤2.75	2.42 ± 0.15	24 months
Tian et al. (24)							2.42 ± 0.18	
SCAAR	Prospective	Exclusion of first-generation DES	SeQuent Please (Braun), IN.PACT Falcon (Medtronic), and Pantera Lux (Biotronik)	Not included	1,154/13,634	<2.5	na	36 months
Silverio et al. (25)								
Funatsu et al. (5)	RCT	POBA	Paclitaxel-coated DEB (SeQuent Please, Braun)	Three lesions (2.9%)	92/41	≥2.0 and <2.75	PCB: 2.04 ± 0.39	6 months
							POBA: 1.99 ± 0.28	
Her et al. (26)	Retrospective	POBA	Paclitaxel-coated DEB (SeQuent Please, Braun)	Not included	49/23	≥2.50 and ≤3.0	PCB: 2.3 ± 0.5	9 months
							POBA: 2.1 ± 0.5	
BIOSCIENCE	RCT	Sirolimus-eluting stent (Orsiro, Biotronik) vs. everolimus-eluting stent (XIENCE Prime/Xpedition, Abbott)			603 (Orsiro)/ 631	<3.0	na	60 months
Iglesias et al. (27)								
CENTURY II	RCT	Sirolimus-eluting (Ultimaster DES, Terumo) vs. everolimus-eluting stent (XIENCE, Abbott)			277 (Orsiro)/248	≤2.5	2.30 ± 0.40	12 months
Wöhrle et al. (28)							2.31 ± 0.42	
XCIENCE V	Retrospective	Everolimus-eluting stent (XIENCE V, Abbott)			838	<2.5	2.55 ± 0.36	12 months
Hermiller et al. (29)								
SPIRIT small vessel	Prospective	Everolimus-eluting stent (XIENCE nano, Abbott)			144	2.25	2.13 ± 0.23	12 months
Cannon et al. (30)								
Kitabata et al. (31)	Retrospective	Everolimus-eluting stent (XIENCE V, Abbott) and Promus (Boston Scientific) vs. sirolimus-eluting stent (Cypher, Cordis)			220 (everolimus)	≤2.5	na	12 months
					423 (sirolimus)			
KAMIR	Prospective	Zotarolimus-eluting stent (Endeavor-ZES, Medtronic) vs. everolimus-eluting stent (XIENCE V, Abbott or Promus			651 (zotarolimus)	≤2.5	na	12 months
Cho et al. (32)					914 (everolimus)			
Nasu et al. (33)	Prospective	Everolimus-eluting stent (Promus, Boston Scientific or XIENCE V Abbott) vs. paclitaxel-eluting stent (Taxus Libertè, Boston Scientific)			264 (everolimus)	<2.5	2.2 ± 0.2 (paclitaxel)	24 months
					245 (paclitaxel)		2.2 ± 0.3 (everolimus)	
Caputo et al. (34, 35)	Retrospective	Zotarolimus-eluting stent (Resolute Integrity, Medtronic)			1,956	≤2.5	2.4 ± 0.4	24 months
Teirstein et al. (35)	Prospective	Everolimus-eluting stent (Promus Element, Boston Scientific)			94	<2.5	2.04 ± 0.26	24 months
Parikh et al. (36)	Retrospective	Zotarolimus-eluting stent (Resolute Integrity, Medtronic)			1,304	>2.25 and ≤2.75	2.6 ± 0.3	36 months
Saito et al. (37)	Prospective	Sirolimus-eluting (Ultimaster DES, Terumo)			70	2.25	1.95 ± 0.28	24 months
Price et al. (38)	Prospective	Zotarolimus-eluting stent (Resolute Onyx, Medtronic)			101	≥2.0 and ≤2.25	1.91 ± 0.26	12 months
Buiten et al. (39)	RCT	Sirolimus-eluting stent (Orsiro, Biotronik) vs. everolimus-eluting stent (Synergy, Boston Scientific) vs. zotarolimus-eluting stent (Resolute Integrity, Medtronic)			525 (sirolimus)	<2.5	2.11 ± 0.28 (sirolimus)	36 months
					496 (everolimus)		2.12 ± 0.28 (everolimus)	
					485 (zotarolimus)		2.11 ± 0.28 (zotarolimus)	
Bartorelli et al. (40)	Prospective	Sirolimus-eluting stent (Orsiro, Biotronik)			245	≤2.75	na	18 months
Guedeney et al. (41)	Prospective	Everolimus-eluting stent (Promus Premier, Boston Scientific)			1,607	<2.5	2.5 [2.3–2-5]	12 months
Jinnouchi et al. (41)	Retrospective	Biolimus-eluting stent (Nobori, Terumo) vs. everolimus-eluting stent (XIENCE V, Abbott or Promus, Boston Scientific)			612 (Biolimus)	2.5 stent size	2.27 ± 0.41 (Biolimus)	24 months
					520 (Everolimus)		2.23 ± 0.39 (Everolimus)	
Funayama et al. (42)	Retrospective		Paclitaxel-coated DEB (SeQuent Please, Braun)	5 (4.5%)	102	<3.0	2.02 ± 0.61	12 months
Sinaga et al. (43)	Prospective		Paclitaxel-coated DEB (SeQuent Please, Braun)	34 lesions (7.2%)	447	<2.8	2.14 ± 0.35	9 months
Onishi et al. (44)	Prospective		Paclitaxel-coated DEB (SeQuent please, Braun)	Not included	52	<2.5	1.93 ± 0.63	8 months
Jim et al. (45)	Retrospective		Sirolimus-coated balloon (ALEX)	na	19	1.5–2.0	1.80 ± 0.25	6 months
Zeymer et al. (46)	Prospective		Paclitaxel-coated DEB (SeQuent Please, Braun)	34 (7.2%)	420	≥2.0 and ≤2.75	2.13 ± 0.34	9 months
Li et al. (47)	Retrospective		na	na	167	<2.8	1.80 ± 0.30	12 months
Yu et al. (48)	Retrospective		Paclitaxel-coated DEB (SeQuent Please, Braun)	1 (0.3%)	327	<2.8	2.43 ± 0.33	10 months
Unverdorben et al. (49)	Prospective		Paclitaxel-coated DEB (SeQuent Please, Braun)	32 (21.1%)	82	2.25–2.8	2.36 ± 0.18	12 months
Kilickesmez et al. (49)	Retrospective	Zotarolimus-eluting stent (Resolute Integrity, Medtronic)			185	<2.5	na	36 months
Ito et al. (50)	Prospective	Everolimus-eluting stent (XIENCE V, Abbott)			681	<2.5	2.24 ± 0.19	12 months
Jim et al. (51)	Retrospective	Zotarolimus-eluting stent (Resolute, Medtronic)			142	≤2.5	2.15 ± 0.21	12 months

PSM, propensity score matched; na, not available.

Data are numbers, or mean ± SD unless otherwise stated; Silverio et al. reported as interval median value.

All analyses were based on previous published studies; thus, no ethical approval or patient consent was required. The investigation is in line with the principles of the Declaration of Helsinki.

### Outcome measures and definitions

The clinical outcomes of the current analysis included trial-defined major adverse cardiac events (MACE), all-cause death, cardiac death, target lesion thrombosis (TLT), target lesion revascularization (TLR), target vessel revascularization (TVR), and myocardial infarction. The pooled analysis was separately performed for all outcomes if available. To achieve a better and more comprehensive comparability of DES vs. DCB in a larger study population, we performed a meta-analytic approach allowing the additional inclusion of single-arm studies. To assess the impact of the remarkably large trial of Silverio et al. (25), a sensitivity analysis by disregarding the concerning study results was performed. Results were reported at the longest follow-up time available and stratified by ≤12 and >12 months, if applicable.

Cardiac death was defined as death of any cardiovascular mechanism, whereas death due to various causes was defined as all-cause death. TLR was defined as any repetitive revascularization within the segment treated with the stent or drug-coated balloon. The definition of myocardial infarction was consistent with the applicable guidelines of myocardial infarction at the time of study. MACE was usually defined as the composite of all-cause mortality, TLR, and MI. TLT was defined as angiographic evidence of thrombosis within the treated lesion.

### Statistical analysis

Continuous variables are expressed as mean and standard deviation (SD). Categorical variables are expressed by means of absolute frequencies and corresponding percentages. A *p*-value of <0.05 was considered statistically significant.

A combination of clinical endpoints and clinical risk factors from single-arm studies followed a meta-analytic approach. Specifically, for the calculation of an overall proportion from studies reporting a single proportion, the inverse variance method was used (*metaprop* function). All effect estimates are presented together with their 95% confidence intervals (CI). To assess the extent of between-study heterogeneity, the *I*² statistic was evaluated leading to the application of a fixed-effects model where *I*² was <40% and a random-effects model otherwise.

In case of studies reporting median and range instead of mean and SD, data were assumed to be normally distributed. As a consequence, the median was assumed to equal the mean, and SD was estimated as range/4 (53).

A comparison of overall measures from single-arm studies between groups of patients treated with different stent types was based on their corresponding 95% CIs, since the application of an appropriate statistical test was not feasible. Non-overlapping CIs may be interpreted as an indication of a non-existing difference (54).

To assess the impact of the remarkably large study of Silverio et al. (25), a sensitivity analysis was conducted by disregarding the concerning study results with respect to the most important clinical endpoints. Forest plots were used for graphical representation of the results.

Furthermore, meta-regression (R package “metafor”) was applied in order to account for possible confounding of the results by different vessel reference diameter if at least 10 studies individually reported on the variable of interest according to the *Cochrane Handbook for Systematic Reviews of Interventions* (55).

Analyses regarding the meta-analytic approach were conducted using the R-Studio software (R version 3.5.1, www.r-project.org). Weighted mean vessel reference diameter and follow-up length with standard deviation were calculated using Microsoft Excel (version 16) for each cohort and compared with the unpaired *t*-test using the *t*-test calculator by GraphPad online.

To determine whether significant publication bias was present, funnel plots were generated.

## Results

A total of 159 potential studies were screened through our searches. After duplicate elimination, 119 articles were further examined. Of these, 37 studies met the inclusion criteria and were included in our analysis (see more in Figure 1). In detail, eight randomized controlled trials, nine comparative studies, and 20 single-arm studies were included, enrolling a total of 31,835 patients with SVD. Among those, 28,147 patients were treated with DES (24 studies) and 3,599 patients with DCB (17 studies). Study characteristics are presented in Table 1. Mean vessel size was 2.36 ± 0.19 mm in the DES cohort and 2.24 ± 0.23 in the DEB cohort (*p* = 0.087). Length of follow-up ranged from 6 to 60 months with a weighted mean of 30.2 ± 11.5 months in the DES cohort. In the DEB cohort, the weighted mean of follow-up length was 18.2 ± 12.2 months ranging from 6 to 36 months. Bail-out stenting rates in patients undergoing DEB ranged between 0.3% and 34.5% and was mainly performed using bare metal stents (BMS) due to dissection or recoil (see more in Table 1).

**Figure 2:**
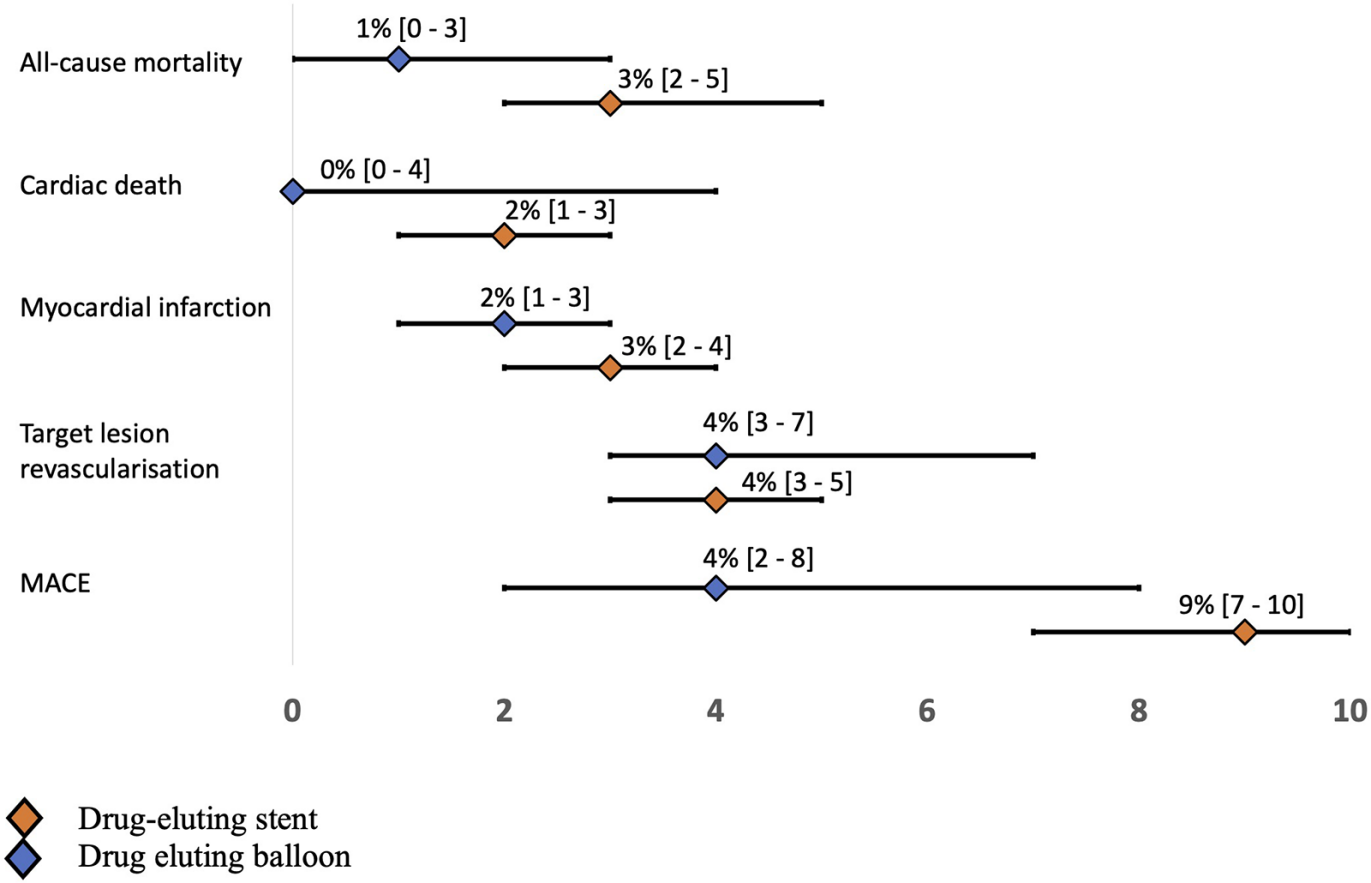
Forest plot results for each outcome.

**Figure 3:**
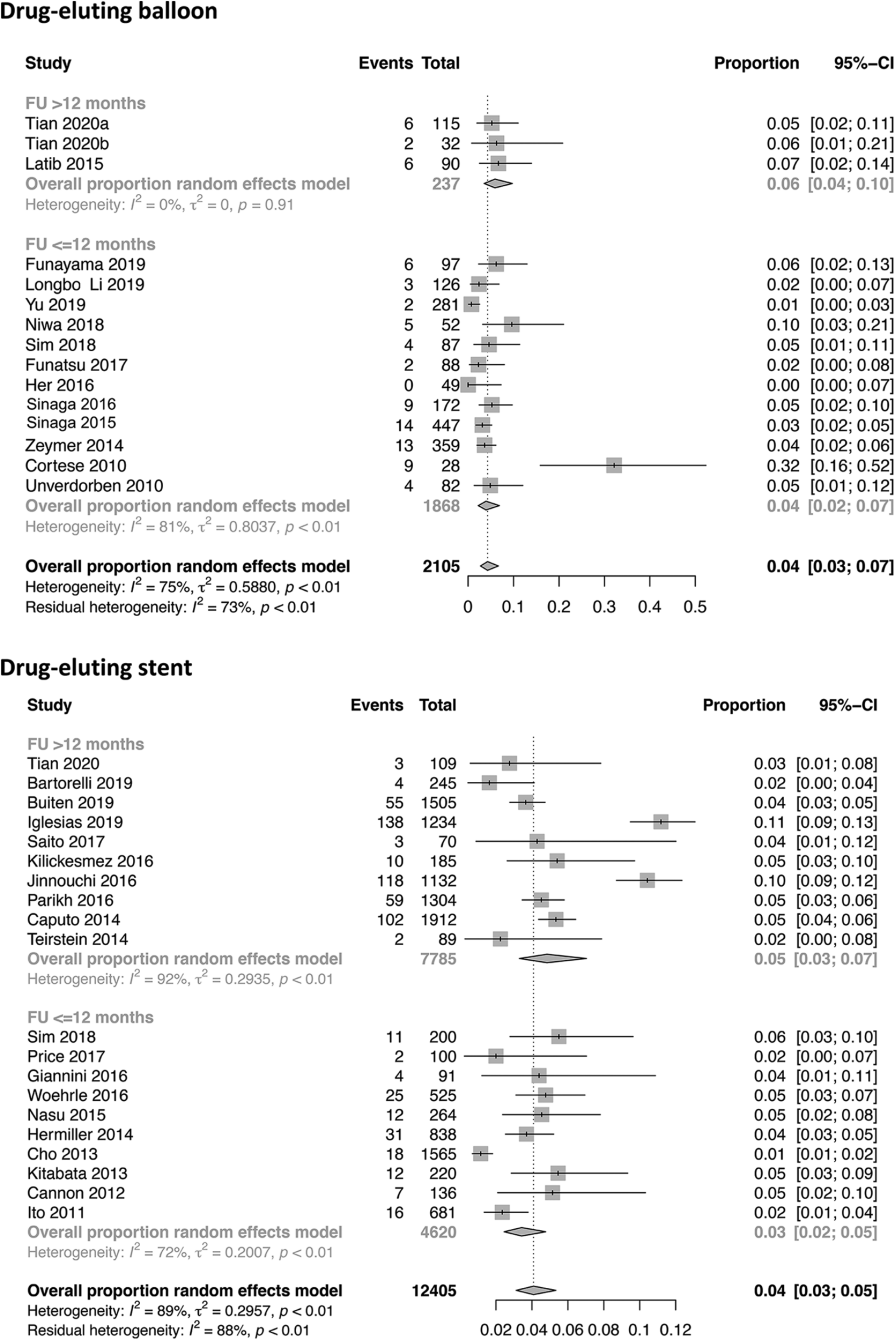
Forest plots of target lesion revascularization in patients undergoing DES or DEB for small-vessel disease stratified by ≤12 and >12 months.

**Figure 4:**
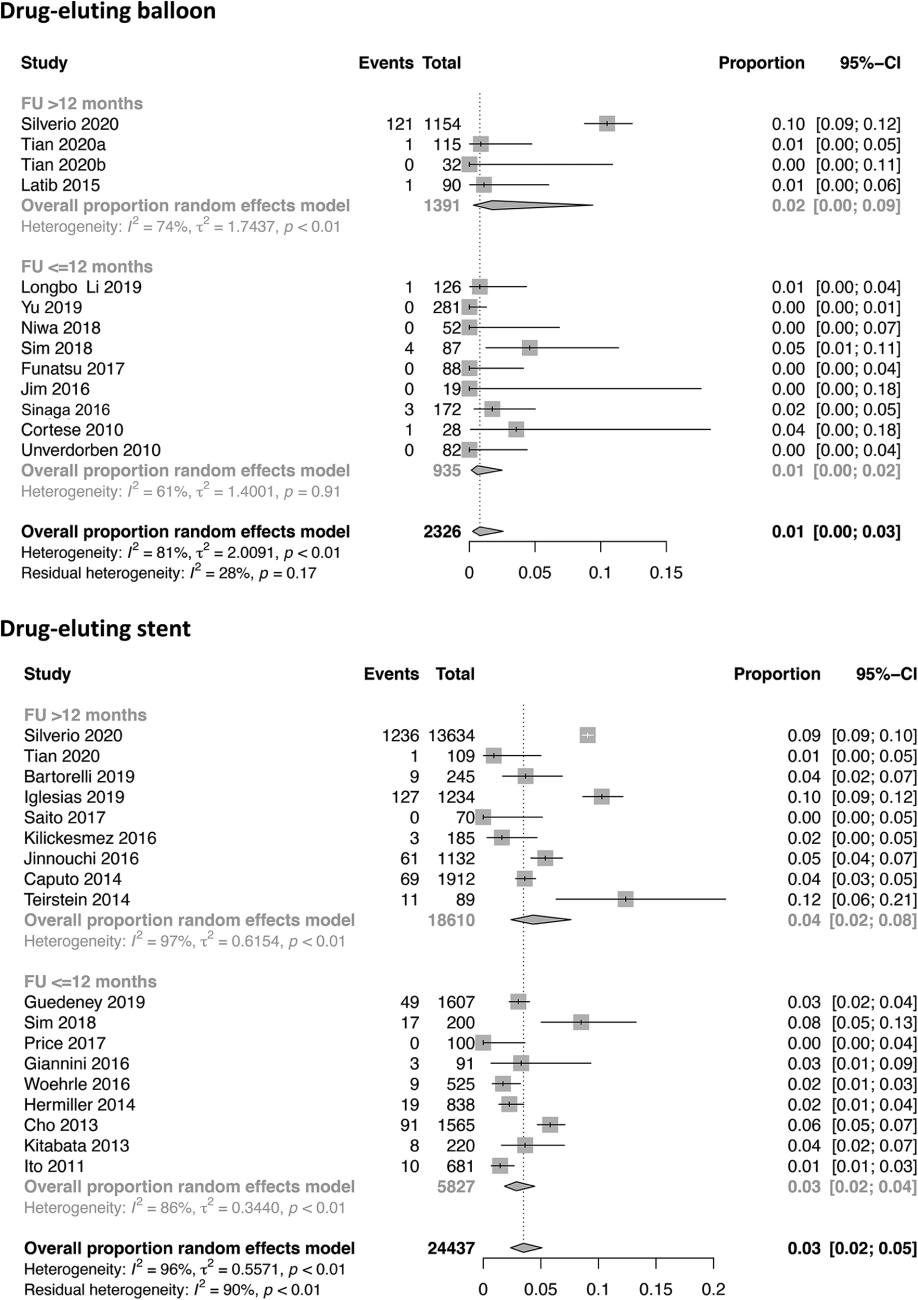
Forest plots of all-cause mortality in patients undergoing DES or DEB for small-vessel disease stratified by ≤12 and >12 months.

Twenty-four studies with a total of 28,147 patients receiving DES and 17 studies with 3,599 patients receiving DCB reported the study outcome of MI. The risk of MI in the SVD population was slightly lower in the DEB group (2%) compared with the DES group (3%) [0.02 (0.01–0.03) vs. 0.03 (0.02–0.04)]. Even when studies were stratified by their follow-up time of ≤12 and >12 months, no significant difference was observed (Figure 2 and Supplementary Figure S1). In terms of TLR [12,405 patients with DES (20 studies) and 2,105 patients with DCB (15 studies)], the incidence was 4% in both treatment strategies [0.04; 95% CI 0.03–0.05 (DES) vs. 0.03–0.07 (DCB); Figure 3]. Additional stratification by follow-up did not show a significant difference (Supplementary Figures S4, S5). All-cause mortality was reported in 18 DES trials (24,437 patients) and 13 DCB trials (2,326 patients). Mortality rate was 3% in the DES group [0.03 (0.02–0.05)] compared with 1% in the DCB group [0.01 (0.00–0.03)] (Figure 4). When studies with a follow-up of up to 12 months were compared, a trend toward a lower all-cause mortality rate was observed in the DEB cohort [0.01 (0.00–0.02) vs. 0.03 (0.02–0.04)]. Cardiac death occurred in 2% of patients treated with DES (20 trials) and 0% of patients treated with DCB [0.02 (0.01–0.03) vs. 0.00 (0.00–0.04)] (Supplementary Figure S2). Fourteen trials with 9,677 patients receiving DES and eight trials with 9,677 patients receiving DCB were included for the combined effect size analysis of the incidence of MACE. MACE rate was 4% in the DCB cohort and lower compared with 9% in the DES cohort [0.04 (0.02–0.08) vs. 0.09 (0.07–0.10)] (Supplementary Figure S3). TVR and TLT were comparable between the DEB cohort and the DES cohort [0.07 (0.03–0.016) vs. 0.06 (0.05–0.08) and 0.01 (0.00–0.02) vs. 0.01 (0.00–0.01)] (Supplementary Figures S4, S5). Funnel plots of all-cause mortality, myocardial infarction, target lesion revascularization, and MACE are displayed in Supplementary Figures S6–S11.

### Sensitivity analysis I: cardiovascular risk factors

**Figure 5:**
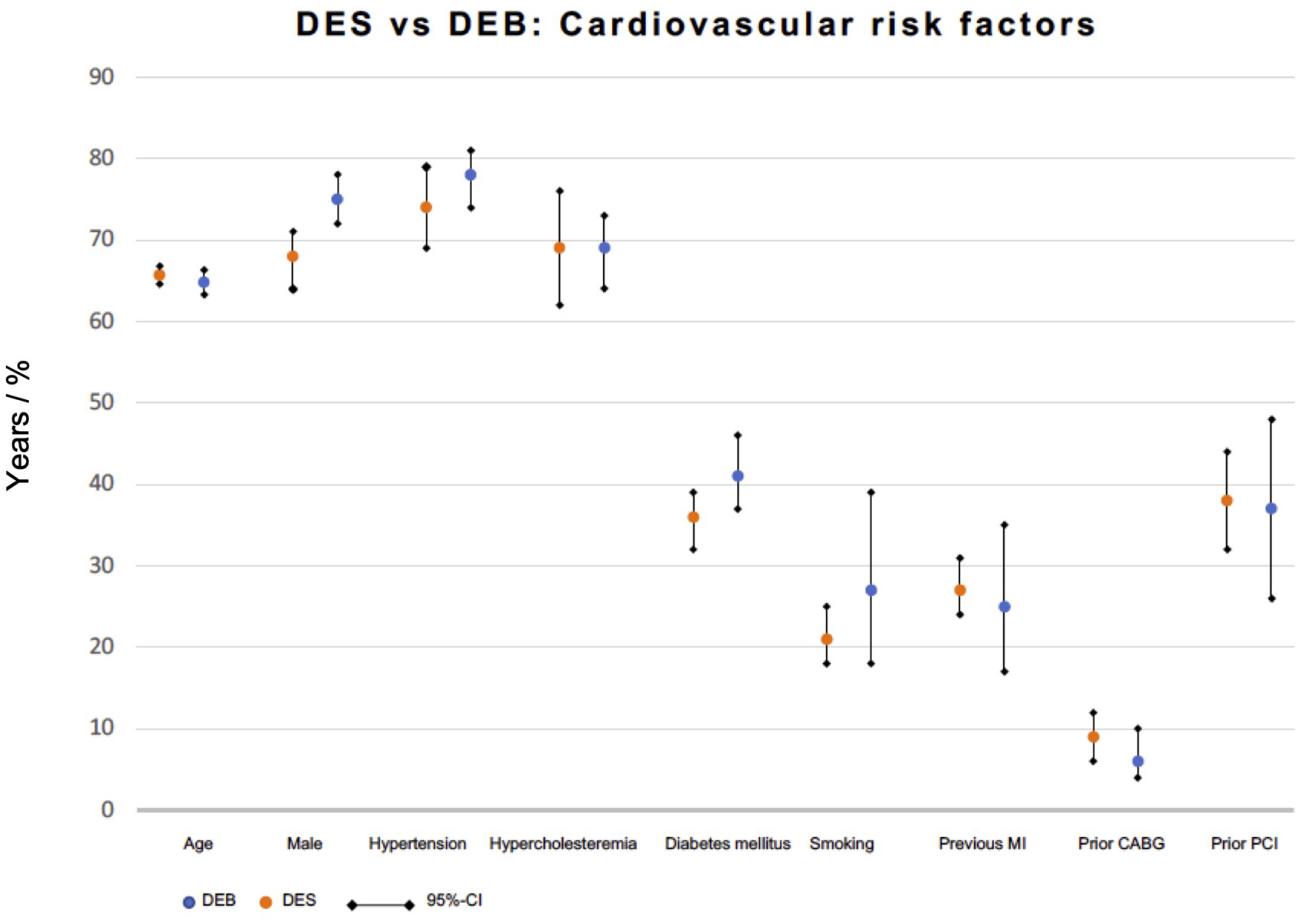
Cardiovascular risk factors in patients undergoing DEB or DES for small-vessel disease.

A sensitivity analysis was performed to evaluate possible risk factors influencing the clinical study endpoints in the two treatment strategies. A total of 28,358 patients out of 24 DES trials and 3,728 patients out of 16 DCB trials were included, and common cardiovascular risk factors were considered. Apart from the variable gender [proportion of males in DES vs. DCB group: 0.68 (0.64–0.71) vs. 0.75 (0.72–0.78)], all other investigated risk factors revealed no significant difference between the DCB cohort and the DES cohort as the respective 95% CI had intersection [age 65.7 (64.6–66.8) vs. 64.8 (63.3–66.3), arterial hypertension 0.74 (0.69–0.79) vs. 0.78 (0.74–0.81), hyperlipidemia 0.69 (0.62–0.76) vs. 0.69 (0.64–0.73), diabetes mellitus 0.36 (0.32–0.39) vs. 0.41 (0.37–0.46), smoking 0.21 (0.18–0.25) vs. 0.27 (0.18–0.39), previous MI 0.27 (0.24–0.31) vs. 0.25 (0.17–0.35), prior CABG 0.09 (0.06–0.12) vs. 0.06 (0.04–0.10), and prior PCI 0.38 (0.32–0.44) vs. 0.37 (0.26–0.48)] (Figure 5 and Supplementary Figures S12–20).

### Sensitivity analysis II: leave-one-out analysis

In order to investigate the influence of the largest included study by Silverio et al., a leave-one-out pooled analysis was performed for the endpoints MI, TLR, and all-cause death, as these events were also investigated in the mentioned trial. It is noticeable that the DEB cohort showed a trend of a significantly lower all-cause mortality after removal of Silverio’s trial [0.01 (0.00–0.02) vs. 0.03 (0.02–0.05)] and a significantly lower mortality rate when studies with a follow-up time of >12 months only were compared [0.01 (0.00–0.03) vs. 0.04 (0.02–0.07). Myocardial infarction [0.02 (0.02–0.03) in the DES vs. 0.02 (0.01–0.03) in the DEB cohort] and target lesion thrombosis [DES cohort 0.00 (0.00–0.01)] did not show a relevant difference to the main analysis. Analysis of the DEB cohort was not possible due to limited study availability (Supplementary Figures S21–S23).

### Meta-regression of reference vessel diameter

Meta-regression of reference vessel diameter was performed for all outcomes reported by at least 10 studies (Table 2). A significant impact was not observed in terms of target lesion revascularization (15 studies; 8,956 patients; *p* = 0.592 in the DES cohort and 14 studies; 2,073 patients; *p* = 0.758 in the DEB cohort), myocardial infarction (17 studies; 9,457 patients; *p* = 0.513 in the DES cohort and 14 studies; 1,966 patients; *p* = 0.700 in the DEB cohort), all-cause (12 studies; 7,354 patients; *p* = 0.125 in the DES cohort and 11 studies; 1,140 patients; *p* = 0.565 in the DEB cohort), or cardiac death (14 studies; 8,326 patients; *p* = 0.960 in the DES cohort and 11 studies; 2,006 patients; *p* = 0.416 in the DEB cohort). In addition, MACE was not affected by the reference vessel diameter in the DES cohort (11 studies; 7,707 patients; *p* = 0.551).

**Table 2:** Meta-regression of vessel diameter in the DEB and the DES cohort for specific outcomes.

Moderator	Estimate	95% CI	*p*-value
Target lesion revascularization			
DEB	0.6277	−1.6662 to 2.9217	0.5917
DES	0.1829	−0.9823 to 1.3480	0.7584
Myocardial infarction			
DEB	−0.5776	−2.3101 to 1.1548	0.5134
DES	0.2756	−1.1272 to 1.6785	0.7002
Death			
DEB	−6.2494	−14.2421 to 1.7432	0.1254
DES	−0.6759	−2.9800 to 1.6283	0.5654
MACE			
DES	0.2904	−0.6651 to 1.2459	0.5514
Cardiac death			
DEB	0.2716	−10.4076 to 10.9508	0.9602
DES	−0.3405	−1.1610 to 0.4800	0.4160

## Discussion

This large meta-analytic approach including 31,835 patients across 37 studies displays the most comprehensive synthesis of data for contemporary percutaneous treatment strategies of small diameter coronary artery stenoses. The main findings of the present study can be summarized as follows: A DCB strategy was at least equivalent to DES therapy in treating SVD in terms of angiographic and clinical endpoints during a follow-up ranging from 6 months to 5 years. The use of DCB was associated with a trend toward lower rates of MI, all-cause death, and MACE compared with DES, however, without reaching statistically significance. The risks of TLR and TLT were similarly distributed in both groups. Reference vessel diameter did not show a significant impact on outcomes in meta-regression analysis.

The prevalence of SVD comprise approximately one-third of patients with symptomatic CAD depending on the definition applied (10, 56, 57). Female gender, diabetes mellitus, and chronic renal failure as well as anatomic subsets such as distal vessel segments and bifurcation lesions were associated with a higher risk for SVD (1, 2, 50, 58, 59). Indeed, we observed a very high prevalence of well-known cardiovascular risk factors in the present analysis. Over one-third of patients suffered from diabetes mellitus, over one-fifth were smokers, and even three-quarters of patients had arterial hypertension. Aside from the high number of affected patients, the fact that even a small ischemic territory can cause limiting angina, impaired quality of life, and malignant ventricular arrhythmias emphasizes the importance of this issue (3, 4, 10, 60).

Despite the development and improvement of many PCI techniques in recent years, small-vessel CAD still remains a challenge for interventional cardiologists (25). Regardless of the treatment type, coronary intervention of lesions located in small vessels is linked to an elevated risk of restenosis and repeat revascularization (61, 62). Common PCI techniques can result in restenosis due to recoil after plain old balloon angioplasty (POBA) or neointimal hyperplasia after stent implantation, which is significantly increased in small caliber vessels compared with larger coronary arteries. This can be explained by their limited ability to adapt neointimal tissue without impeding blood flow (25, 62, 63). In a large study with over 10,000 patients treated with stent implantation in small vessel, reference vessel diameter was demonstrated to be the most relevant predictor of angiographic restenosis with a 60% higher risk of restenosis for each decrease in reference vessel diameter by 0.50 mm (7).

Although second-generation DES are known to reduce the risk for restenosis in the overall CAD population compared with POBA and BMS implantation, their efficacy is still limited in small coronary arteries (64, 65). *Per se*, DES are as effective in small as in large vessels; however, the resulting late lumen loss occupies a higher percentage of the respective vessel diameter, resulting in elevated rates of in-stent restenosis and clinical events (12, 66).

In the past decade, the development and widespread use of DCB offered a promising treatment option for patients with SVD. DCBs have already been shown to be a suitable option for treatment of in-stent restenosis after BMS or DES implantation (class I level A) (67–72). The technique relies on the rapid and homogenous application of antiproliferative drugs into the vessel wall without using permanent implants (73, 74).

Nowadays, DES is the device of choice in most PCIs; however, in patients with SVD, DCB offers an attractive alternative with some potential advantages over DES (12, 75). The potential risk of stent thrombosis representing the most feared complication of DES, reduced duration of dual antiplatelet therapy of only 4 weeks, and the lack of a permanent vascular cage leftover inside the coronary circulation may represent additional features favoring DCB over DES. Moreover, the smaller profile compared with DES provides easier access to complex lesions, particularly in small vessels (75).

On the other hand, it has similar disadvantages to POBA such as following elastic recoil or dissections, which occasionally necessitate bail-out stenting (76, 77). Furthermore, the shortened balloon inflation time and the scour blood flow raise doubts about a sufficient delivered drug amount to the vessel wall and a DES-equivalent drug maintenance at the target lesion over time (57).

The clinical proof of concept when using a DCB strategy in the treatment of SVD has initially been demonstrated in several non-randomized studies and registries (78–82). Subsequently, several randomized clinical trials comparing DCB with balloon angioplasty (5), BMS (83), and DES were performed (12, 17, 18, 23, 84). Especially early trials and meta-analyses comparing DCB with DES failed to show equivalent results to DES regarding angiographic and clinical endpoints during PCI when a DCB strategy was used (6, 17). However, the lack of superior efficacy of DCBs was primarily attributed to the DCB’s characteristics, such as the excipient, the drug transfer rate, an insufficient implantation technique, and geographic mismatch (12, 17, 85). This is demonstrated by the contradictory results of the PICCOLETO I and PICCOLETO II studies, which can be explained by significant improvements in DCB technology (17, 86). The PICCOLETO I trial using an early-generation DCB was stopped after randomization of only 60 patients due to the superiority of DES in terms of lower rate of restenosis at 6-month angiographic follow-up (25). Contrarily, the PICCOLETO II study found a novel DCB to be superior to DES in terms of late lumen loss and comparable regarding clinical outcomes (86). This is strengthened by several previous studies demonstrating non-inferiority of DCB compared with DES. Accordingly, the BELLO trial, which enrolled 182 patients with lesions located in vessels of <2.8 mm showed significantly less late lumen loss in patients treated with DCB compared with DES (18). The rate of MACE was similar in both groups at 6 months and even lower in the DCB group at 3-year follow-up (25). In the RESTORE SVD study including 230 patients with SVD between ≥2.25 and ≤2.75 mm, PCI with DCB was non-inferior to 9-month in-segment percentage diameter stenosis and showed a comparable 1-year rate of target lesion failure (84). Recently, long-term data from the BASKET-SMALL II trial including 758 patients with *de novo* lesions in coronary vessels of <3 mm have been published and strengthened the role of DCB as a promising option in the treatment of SVD (23). The study results indicate continued efficacy and safety of DCB vs. DES in the treatment of SVD up to 3 years.

This is the largest analysis up to date directly comparing the efficacy of DCB and DES strategy in patients with SVD and may clarify the ideal strategy for treating this patient population. Compared with the latest studies focusing on this issue, roughly 10 times more patients were included in the present analysis underlining the strength of our study. This was enabled by a meta-analytic approach additionally including single-arm studies reporting on DES or DEB interventions for SVD only. Consequently, precise estimators with narrow 95% CIs can be obtained from an even larger dataset. In contrast, smaller analyses with a limited number of studies and consequently smaller event rates are at risk to be underpowered for identification of smaller significant differences between groups.

We demonstrated at least non-inferiority of a DCB strategy compared with a DES strategy in terms of angiographic and clinical outcomes in the treatment of SVD. The incidence of TLR, TVR, TLT, and cardiac death was consistent between DES and DEB, whereas DCB showed a trend of lower MI and MACE rates as well as all-cause mortality.

Undoubtedly, the study of Silverio et al. certainly accounts for a large part of our analysis (11). This observational multicenter study from Swedish Coronary Angiography and Angioplasty Registry (SCAAR) including 14,788 patients who underwent elective or emergency percutaneous coronary intervention for *de novo* lesions in small vessels, defined by a device diameter of ≤2.5 mm, suggested that DCBs are not an equally effective alternative to DES for percutaneous treatment of SVD. A strategy with DCBs was associated with a significantly higher risk for restenosis up to 3 years and a similar risk for target lesion thrombosis, MI, and all-cause death in comparison with DES. However, even after excluding the study results of this remarkably large trial in a sensitivity analysis, a non-inferiority of DCBs in the treatment of SVD persisted.

Some important limitations may have influenced the study results by Silverio et al. (25, 87). Among others, diabetes mellitus displaying an established predictor of the studies’ primary outcome restenosis was more prevalent in patients receiving DCBs compared with DES (7, 87). Second, no data were reported if routine or adequate pre-dilatation was performed, having a potential impact on the long-term success of DCB application (87, 88). Adequate lesion preparation with successful pre-dilatation to avoid elastic recoil and flow-limiting dissections is usually an essential preceding application of DCBs (89). Moreover, the drug uptake may be enhanced by adequate pre-dilatation prior to DCB application by creating microdissections in the vessel wall and thus enhancing drug transport through the intima and media layers (75, 90). Silverio et al. inferred the vessel size by the device size implanted and not by visual estimation as in most previous studies, which may result in under- or oversizing of the treated vessel, which could influence the study results especially in patients with SVD. Finally, the study endpoint of angiographic restenosis was evaluated following clinically driven repeat angiography and not by routine angiographic follow-up. Thus, the true rate of restenosis could be underestimated.

Our results are in line with recent meta-analyses focusing on this important issue (59, 75, 77, 91–93). The conflicting results of former studies can be at least partly attributed to differences in the definition of small vessels, devices implanted, implantation technique, use of pharmacological therapies and outcomes evaluated, and small sample sizes (87). This heterogeneity of previous trials in various aspects such as pretreatment rates and device types used may also have influenced the study results of the present analysis.

It should be emphasized that there is no standardized definition of small coronary vessels used in literature up to date. While some trials such as the BASKET-SMALL 2 study defined SVD by a vessel diameter of <3.0 mm, other trials such as the PICCOLETO or the RESTORE SVD China trials used a diameter cut-off of ≤2.75 mm or even ≤2.5 mm as in the SCAAR study (25, 78, 84, 86). However, it should be considered that vessel diameter constitutes a continuous variable and should be regarded as such when analyzing its impact on prognosis including the risk of in-stent restenosis or stent thrombosis (10). Therefore, in the present analysis, we set a vessel diameter threshold of ≤3.0 mm to enable the most comprehensive analysis of treatment strategies in SVD. Furthermore, we demonstrated that the different vessel diameters were not associated with the occurrence of adverse events. Nevertheless, it is possible that larger vessel diameter may benefit more from a DES strategy compared with smaller vessel sizes.

Mostly all included trials used paclitaxel-coated balloons, while the DES type used varied. In contrast to paclitaxel-eluting balloons, limus-coated balloons are comparatively underdeveloped, and clinical data are scarce. Available evidence supporting the efficacy of DEB in the coronary territory was predominantly obtained from DCB eluting paclitaxel (12, 68, 94). In new-generation DES, limus-type drugs have displaced taxane devices due to superior safety and efficacy (68). However, when eluted from a DEB, limus drugs do not exhibit high lipophilicity and have difficulties to effectuate sufficient tissue penetration and retention (12, 94). Recent studies with enhanced DEB technology have shown promising results for limus-coated balloons, too, but studies comparing limus-coated and paclitaxel-coated balloons and limus-coated balloons and new-generation DES in SVD are urgently required to confirm our study results (12, 95–98).

The present data suggested that DEB representing a concept of “leaving nothing behind” may be particularly alternative or even superior to DES in the treatment of SVD. However, larger randomized trials with longer follow-up are required to confirm our findings and to verify the reliability of DCB in SVD. Further follow-up may result in DCB favoring results considering a stent-related adverse event rate of about 2% per year (99).

### Limitation

Our results should be interpreted in view of the following limitations, including well-known confinements of meta-analyses. However, a summary measure from the available trials may be the best estimate of the impact of an intervention.

First, the studies included in the present analysis had differed in clinical and methodological characteristics without standardized criteria.

Second, the definition of SVD ranged from ≤2 to ≤3 mm creating heterogeneity. The fact that vessel size has been shown to inversely correlate with the risk of restenosis after PCI underlines the need for a uniform definition of SVD (63, 100).

Third, the use of different device types both for DES and DCB and different pretreatment rates could be an important source of heterogeneity which may affect the results. However, we only included studies with newer-generation DES as they have shown lower rates of MACE and stent thrombosis and are associated with improved outcomes in SVD compared with older-generation DES (38, 41, 101). Except of one small single-arm study, all included trials used paclitaxel-eluting balloons, while the DES type used varied. Fourth, the analysis was performed using published data and not patient-level data. Consequently, analysis of the impact of baseline clinical and angiographic variables on treatment effects such as an identification of potential differences regarding available treatments in specific patient subgroups (e.g., impact of vessel size on treatment effect) was limited to meta-regression analysis of the reported data. Thus, the findings need to be considered average effects.

Fifth, the length of clinical follow-up varied from 6 months to 5 years across the included studies. Moreover, the follow-up periods of some studies were short. A longer clinical follow-up in all studies would be essential to finally confirm the safety and efficacy of DCB as compared with DES in the long term.

Sixth, there is a lack of routine angiographic follow-up. Most studies reported about clinically driven TLR, which potentially underestimates the correct rate of restenosis. However, routine angiographic follow-up of patients undergoing PCI is not recommended by current guidelines.

Seventh, some important prognostic indicators such as stent thrombosis or major bleeding were not evaluated due to the limited number of included studies evaluating these endpoints. However, rates of major bleeding were similar between DES and DCB in the BASKET-SMALL 2 trial (23). Moreover, the limited number of studies evaluating some endpoints such as cardiac death and the small event rate for these outcomes reduces the likelihood to detect a statistically significant finding between DES and DEB implantation.

Eighth, differing cohort sizes could have affected our results and may be explained by different frequencies of each treatment for SVD.

Ninth, data about the prescribed antiplatelet therapy and its duration were not available for all included studies. Furthermore, we included an all-comers population with different recommendations for antiplatelet therapy. Therefore, an influence of this aspect on our study results cannot be completely ruled out.

Finally, our meta-analytic approach compares studies, which did not primarily compare DES and DEB. Consequently, the presented estimators are not adjusted, and risk-of-bias assessment was not feasible due to the use of single-arm studies. However, this can be considered a strength resulting in large datasets of patients undergoing DES or DEB for SVD. The tendency toward a higher risk in the DES group might be conditioned by a greater inherent risk in patients receiving DES in non-randomized trials, e.g., due to contraindications to DEB strategy. Nevertheless, our study results are in line with recent meta-analysis focusing on this issue and only including randomized trials (59, 91).

## Conclusion

To the best of our knowledge, this comprehensive analysis is the largest comparing contemporary treatment options in small-vessel CAD. Our results suggest that DCB is non-inferior to DES in terms of clinical and angiographic endpoints in lesions of small coronary arteries and represents an effective or even favorable alternative to stent strategy. Compared with DES, PCI of small vessels using DCB was associated with numerically lower rates of MI, all-cause death, and MACE. Therefore, DES may be waived in small coronary arteries when PCI is performed with DCB.

## Data Availability

The original contributions presented in the study are included in the article/**Supplementary Material**, further inquiries can be directed to the corresponding author.
