# Supplementary material for: Drug-coated balloon: an effective alternative to stent strategy in small-vessel coronary artery disease—a meta-analysis
Source: Front Cardiovasc Med. 2023 Aug 21;10:1213992. doi: 10.3389/fcvm.2023.1213992 (PMC10475729; doi:10.3389/fcvm.2023.1213992)
Supplement: Supplementary file 1 [file Datasheet1.pdf]

Supplements

Table S1 NIH rating of the included studies	
Study	NIH
Piccoleto<br>Cortese et al. 2010	Good
BELLO<br>Latib et al. 2012	Good
Giannini et al. 2017	Good
Sim et al. 2018	Good
Sinaga et al. 2016	Good
BASKET-SMALL 2<br>Jeger et al. 2018	Good
RESTORE SVD China<br>Tian et al. 2020	Good
SCAAR<br>Silverio et al. 2020	Good
Funatsu et al. 2017	Good
Her et al. 2016	Good
BIOSCIENCE<br>Iglesias et al. 2019	Good
CENTURY II<br>Wöhrle et al. 2016	Good
XCIENCE V<br>Hermiller et al. 2014	Good
SPIRIT Small Vessel<br>Cannon et al. 2012	Fair
Kitabata et al. 2013	Good
KAMIR<br>Cho et al. 2014	Good

Nasu et al. 2016	Good
Caputo et al. 2014	Good
Teirstein et al. 2015	Fair
Parikh et al. 2016	Good
Saito et al. 2019	Good
Price et al. 2017	Good
Buiten et al. 2019	Good
Bartorelli et al. 2019	Good
Guedeney et al. 2019	Good
Jinnouchi et al. 2016	Good
Funayama et al. 2020	Fair
Sinaga et al. 2015	Good
Onishi et al. 2019	Good
Jim et al. 2016	Fair
Zeymer et al. 2014	Good
Longbo Li et al. 2019	Fair
Yu et al. 2019	Good
Unverdorben et al. 2010	Good
Kilickesmez et al. 2016	Good
Ito et al. 2011	Good
Jim et al. 2014	Fair
References of the rated studies are displayed in the manuscript.	

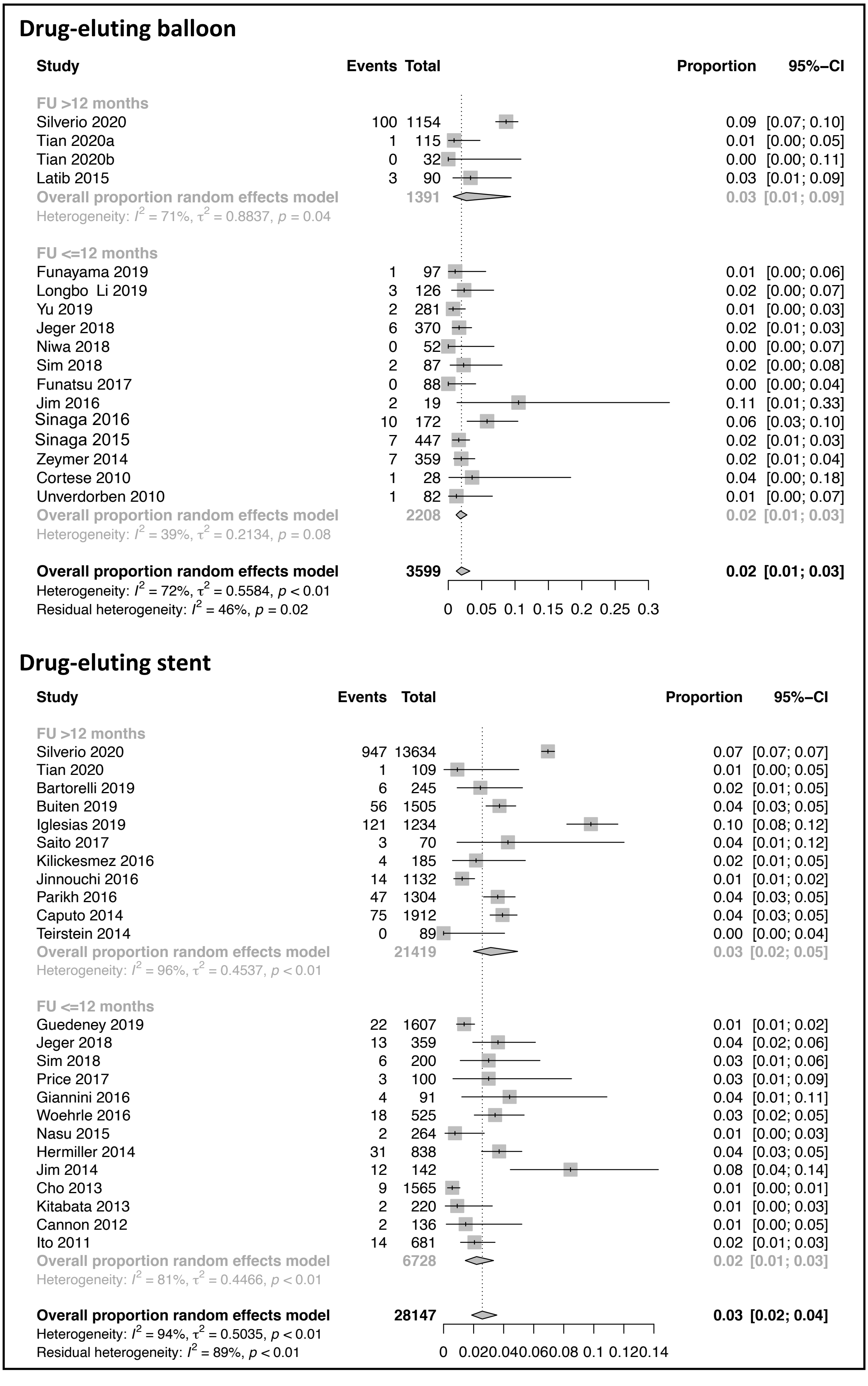

Figure S1 Forest plots of myocardial infarction in patients undergoing DES or DEB for small vessel disease stratified by ≤12 and >12 months

DEB: Drug-eluting stent. DES: Drug-eluting balloon

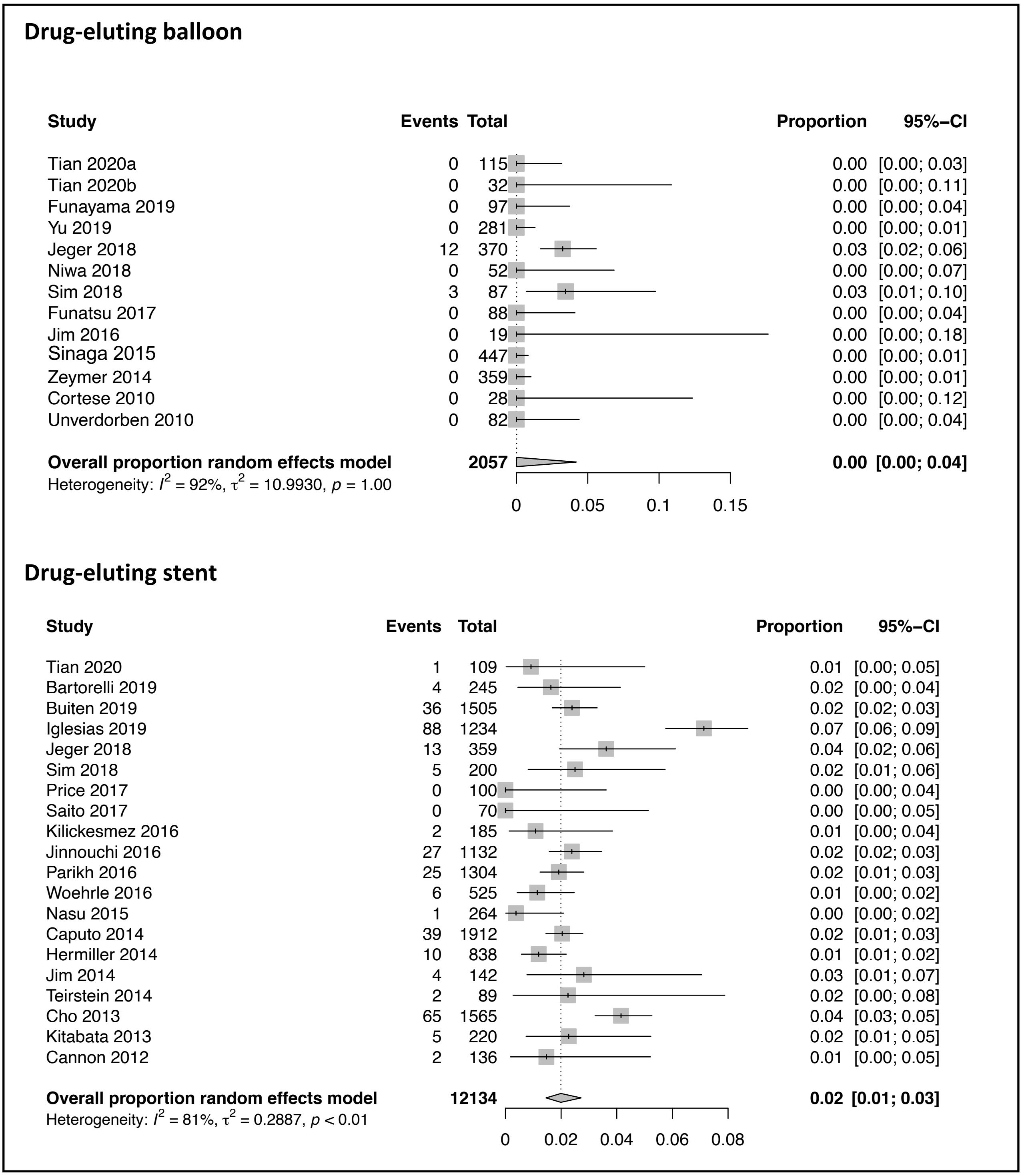

Figure S2 Forest plots of cardiac death in patients undergoing DES or DEB for small vessel disease

DEB: Drug-eluting stent. DES: Drug-eluting balloon

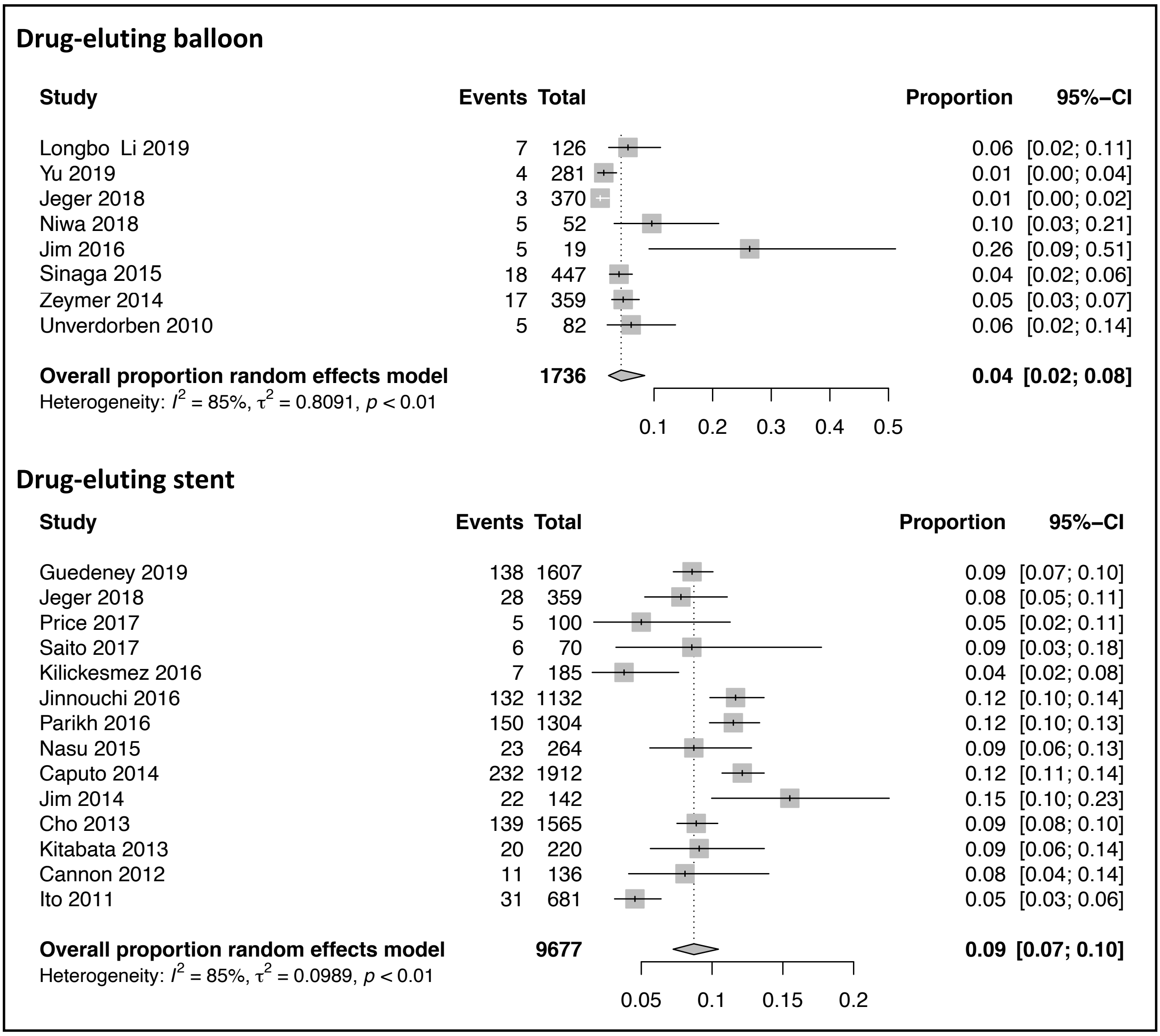

Figure S3 Forest plots of MACE in patients undergoing DES or DEB for small vessel disease

DEB: Drug-eluting stent. DES: Drug-eluting balloon

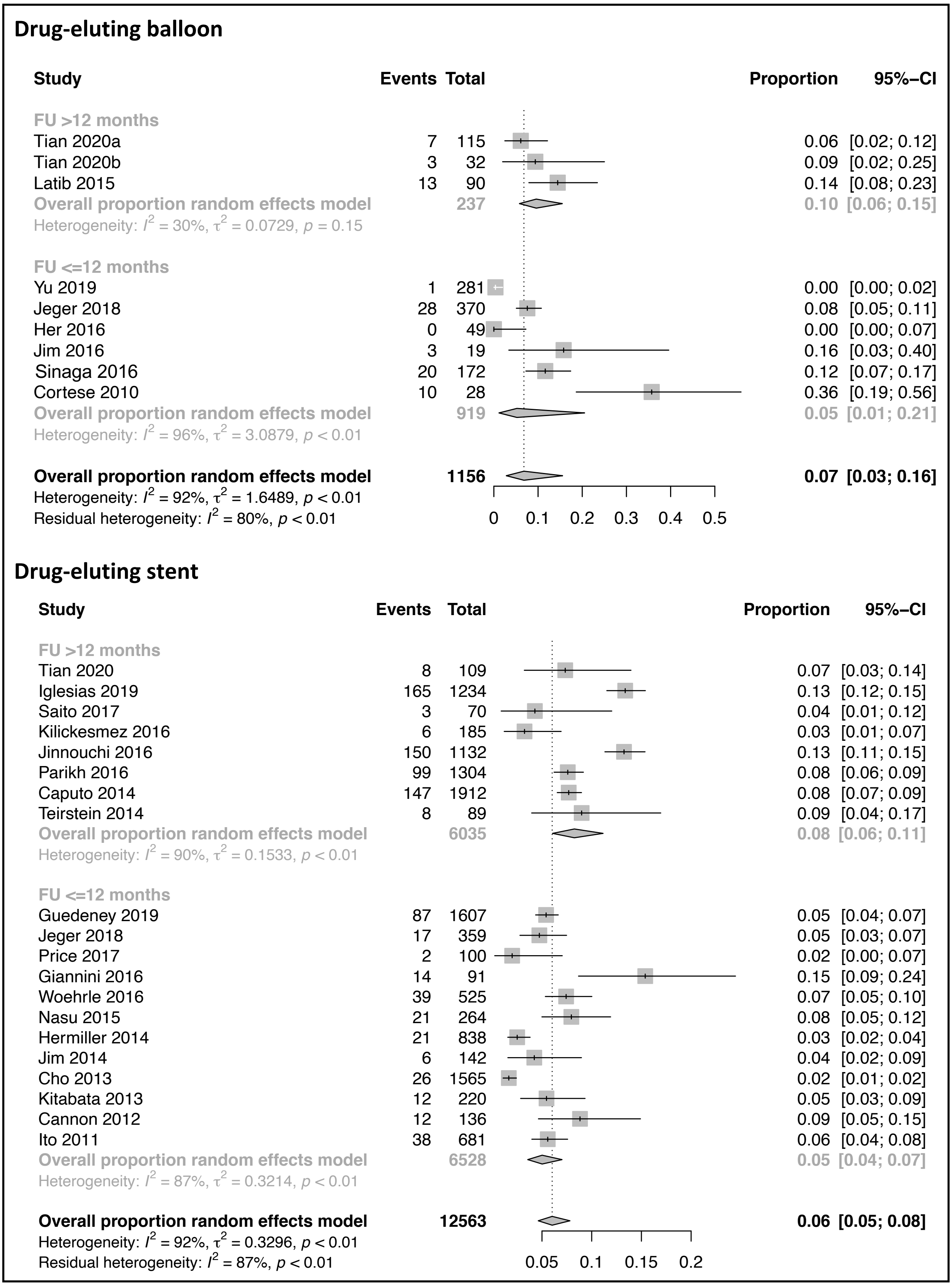

Figure S4 Forest plots of target vessel revascularisation in patients undergoing DES or DEB for small vessel disease stratified by <12 and ≥12 months

DEB: Drug-eluting stent. DES: Drug-eluting balloon

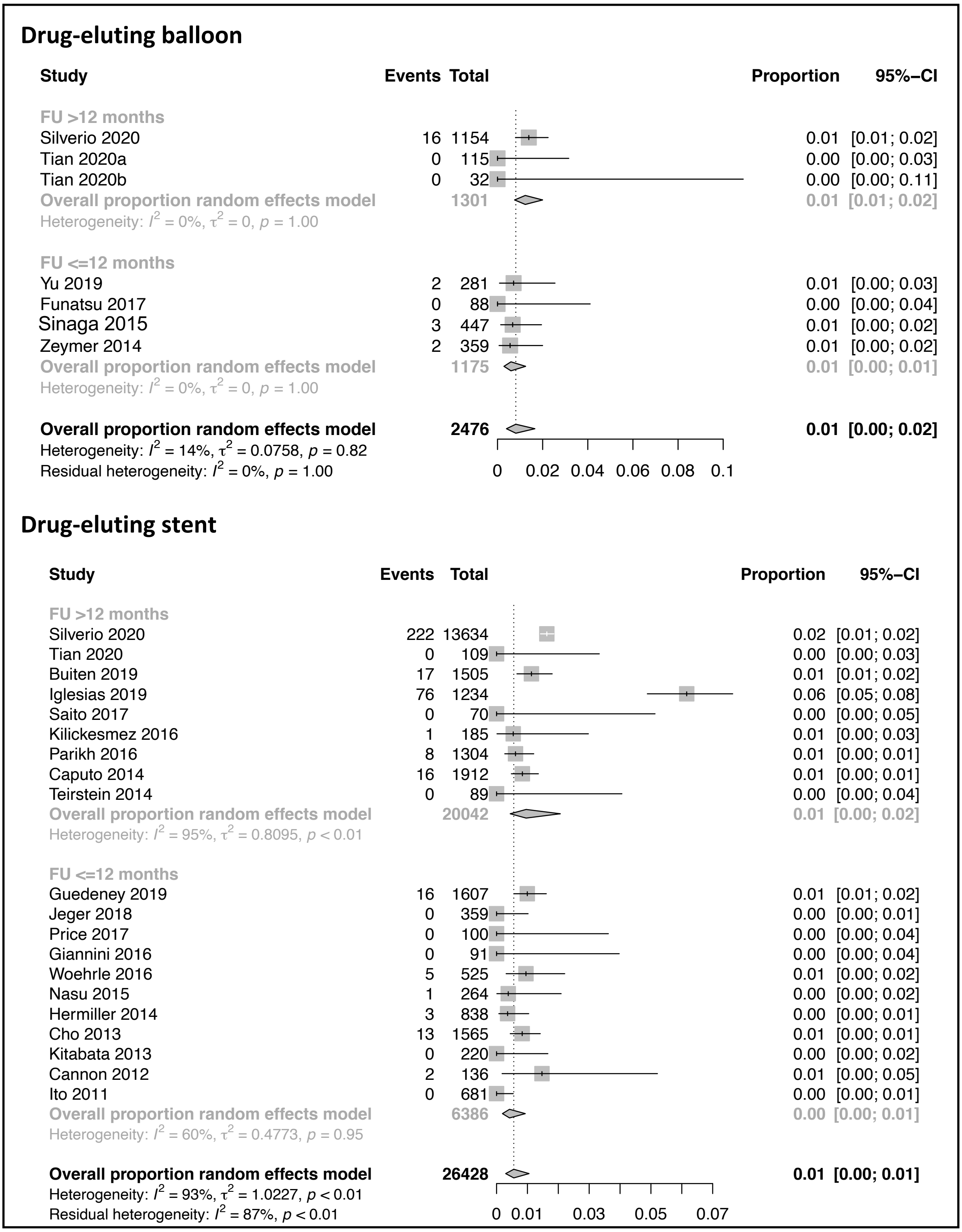

Figure S5 Forest plots of target lesion thrombosis in patients undergoing DES or DEB for small vessel disease stratified by <12 and ≥12 months

DEB: Drug-eluting stent. DES: Drug-eluting balloon

DEB studies

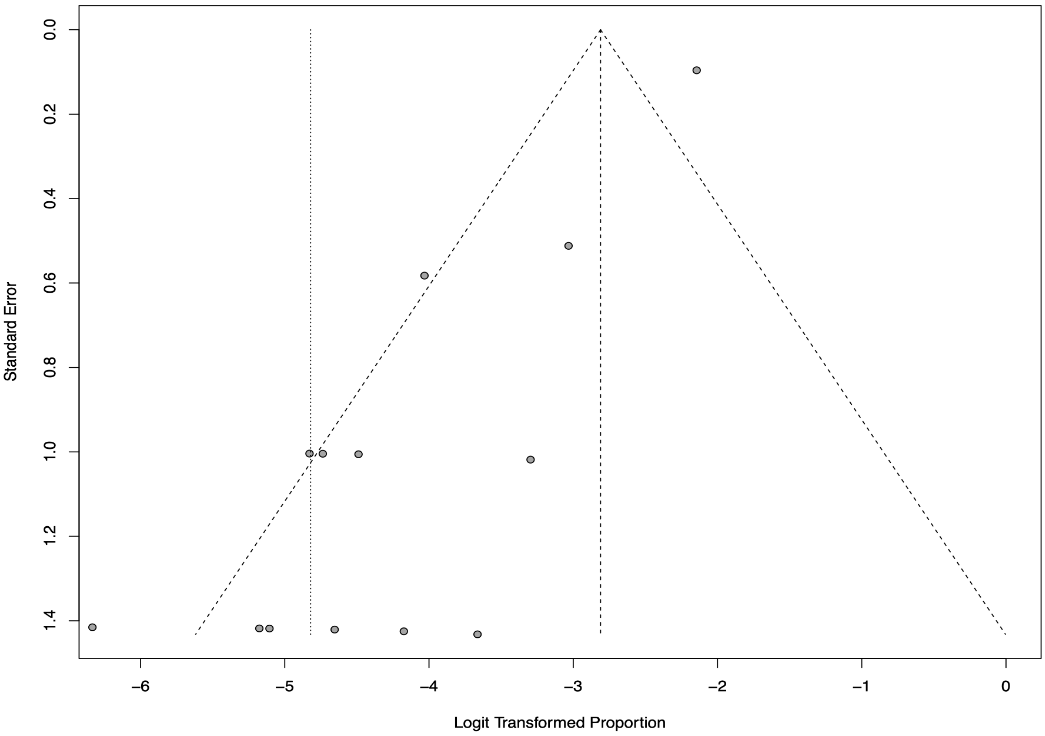

DES studies

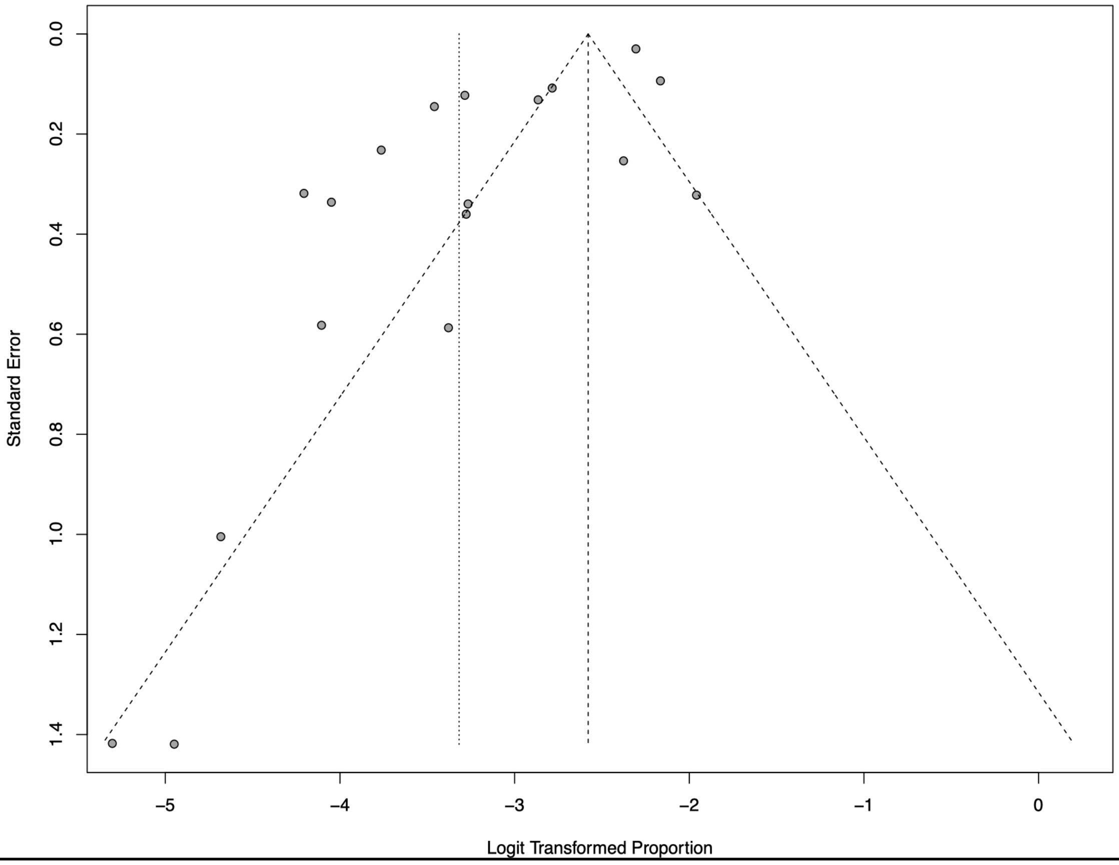

Figure S6 Funnel plots of studies reporting on all-cause mortality

Drug-eluting stent. DES: Drug-eluting balloon

DEB studies

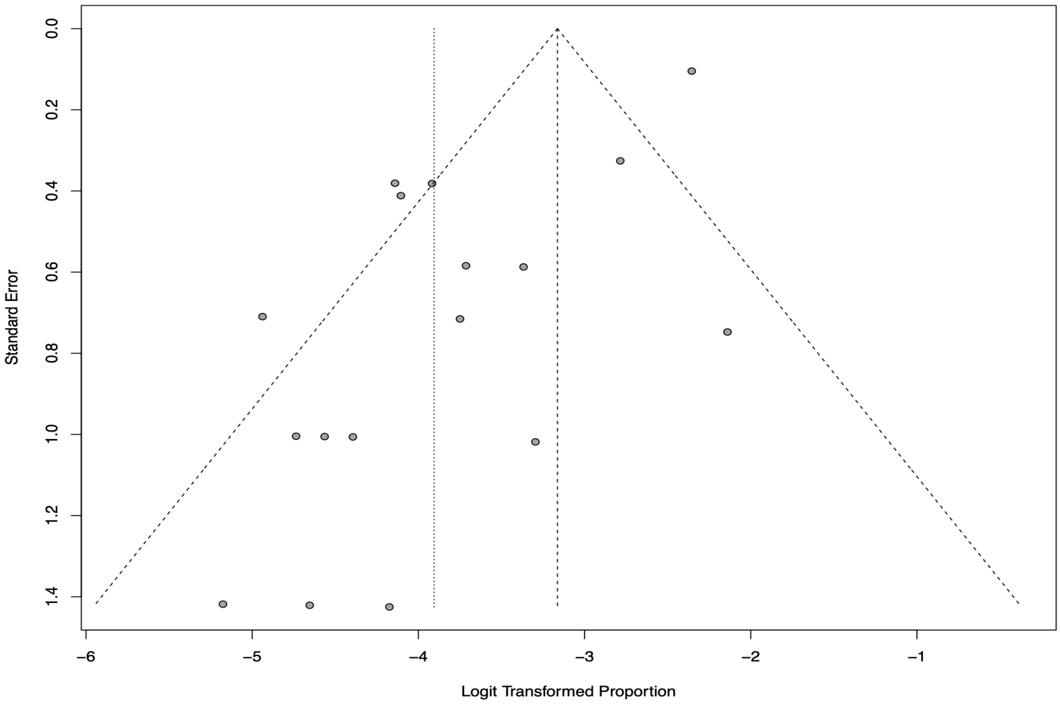

DES studies

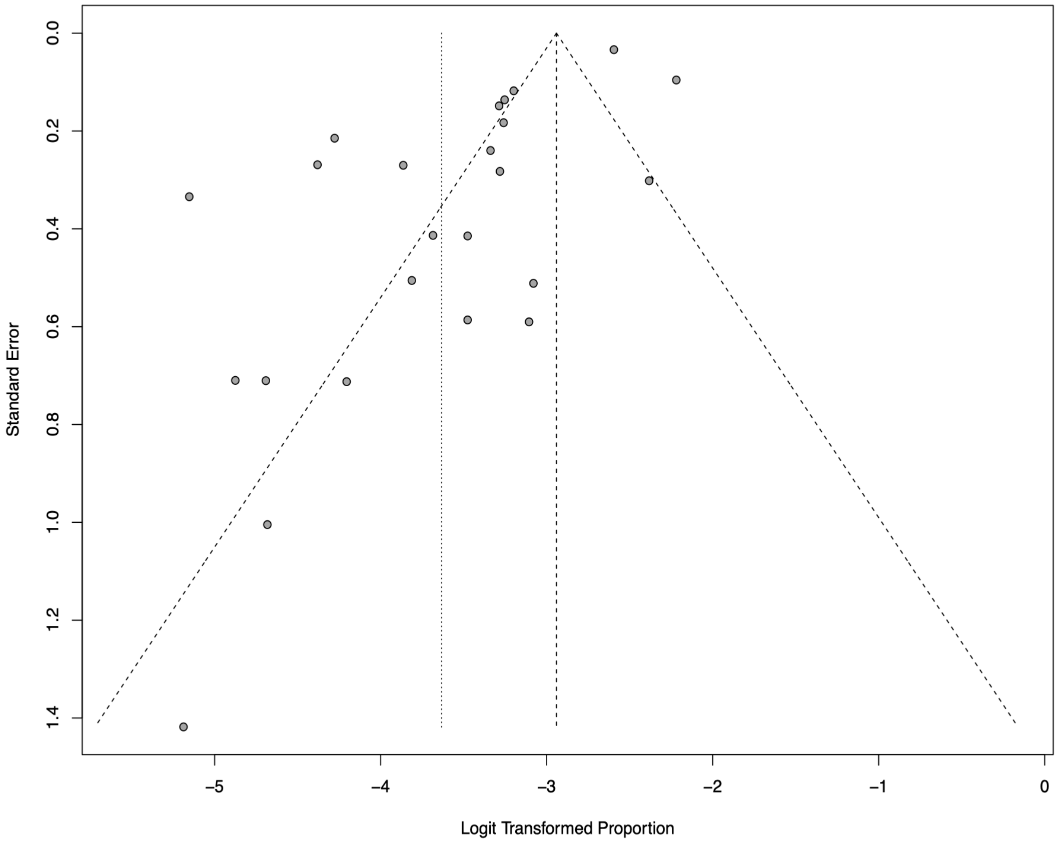

Figure S7 Funnel plots of studies reporting on myocardial infarction

Drug-eluting stent. DES: Drug-eluting balloon

DEB studies

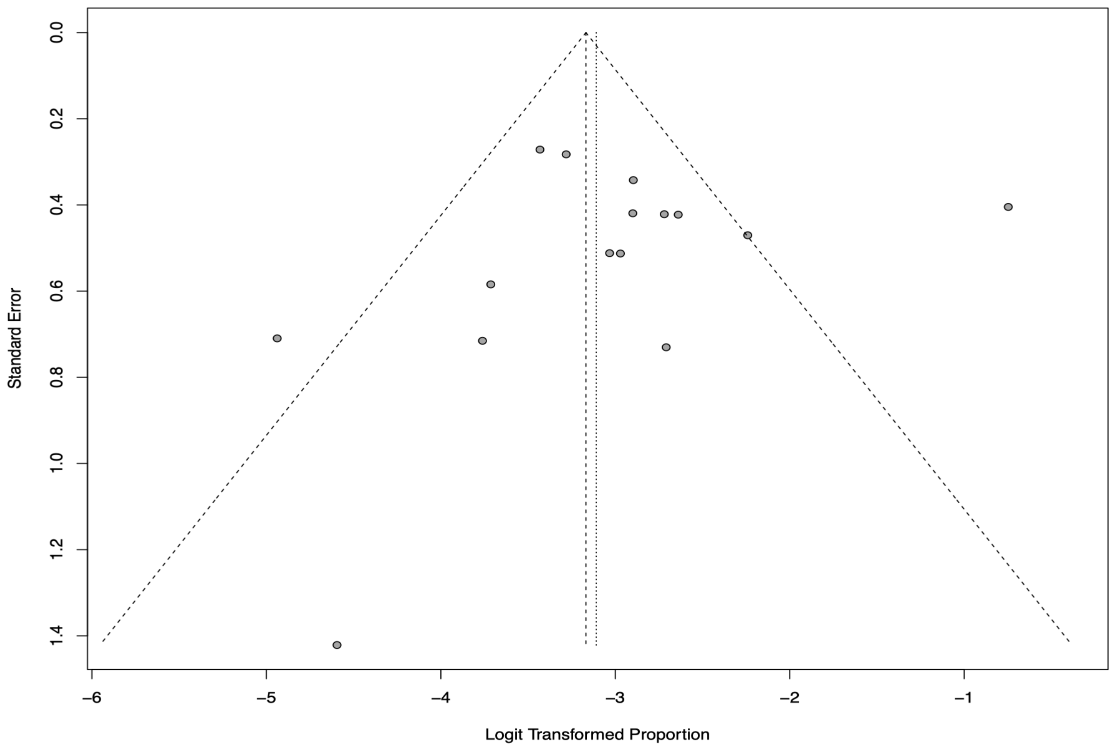

DES studies

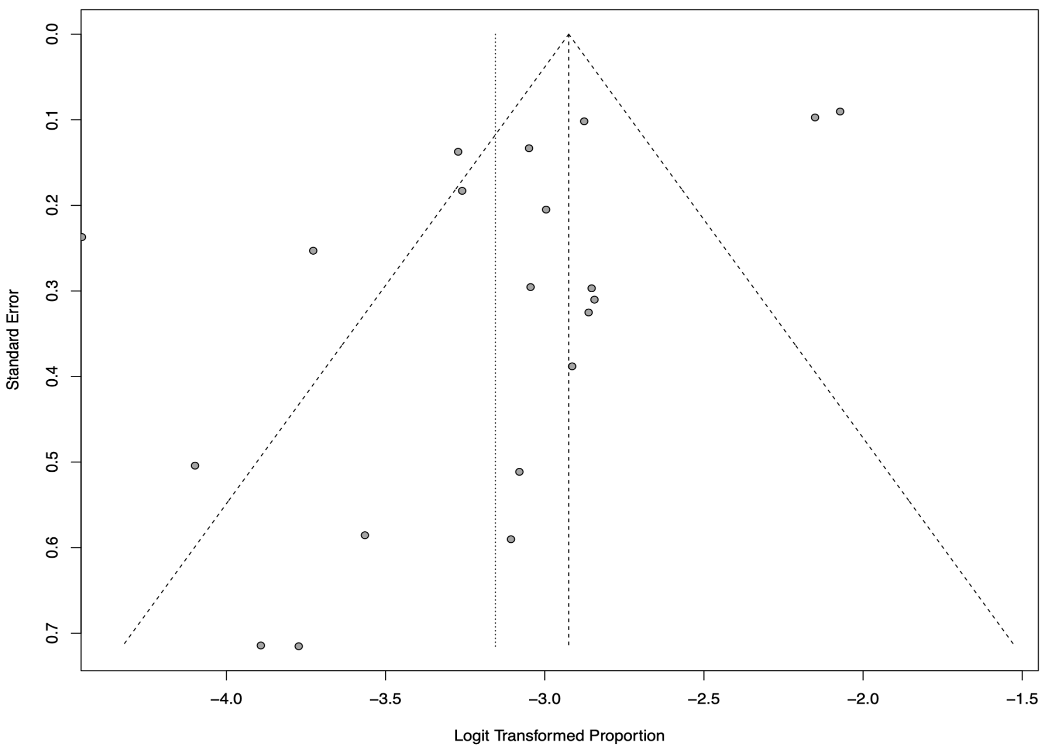

Figure S8 Funnel plots of studies reporting on target-lesion revascularization

Drug-eluting stent. DES: Drug-eluting balloon

DEB studies

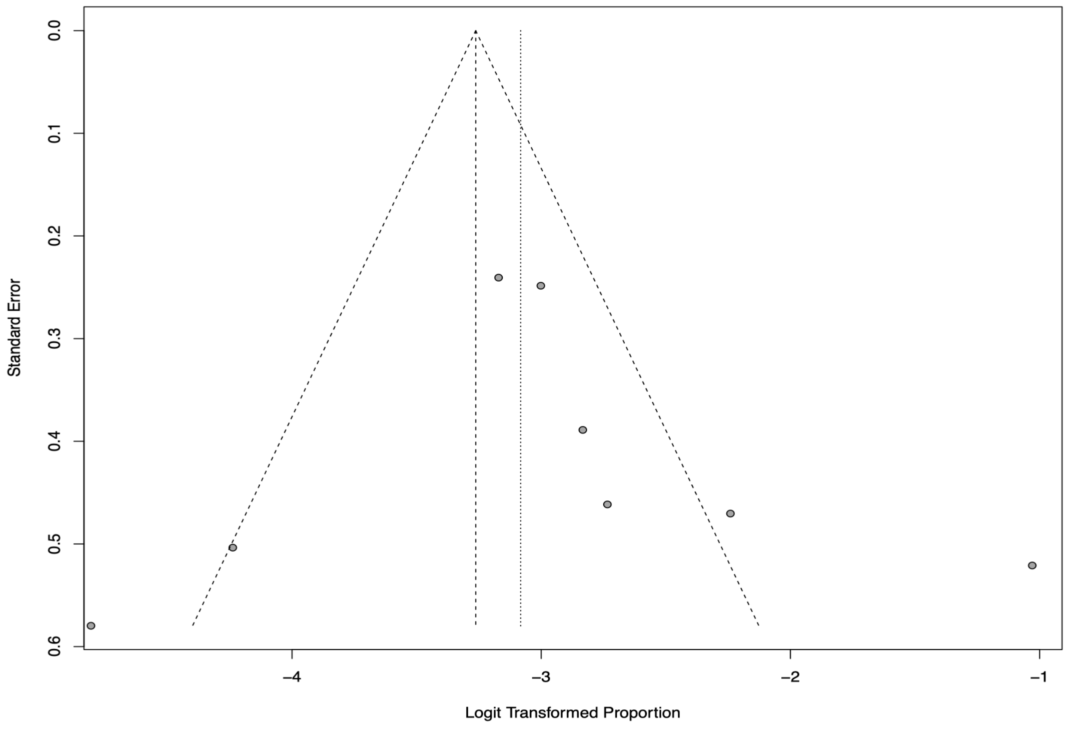

DES studies

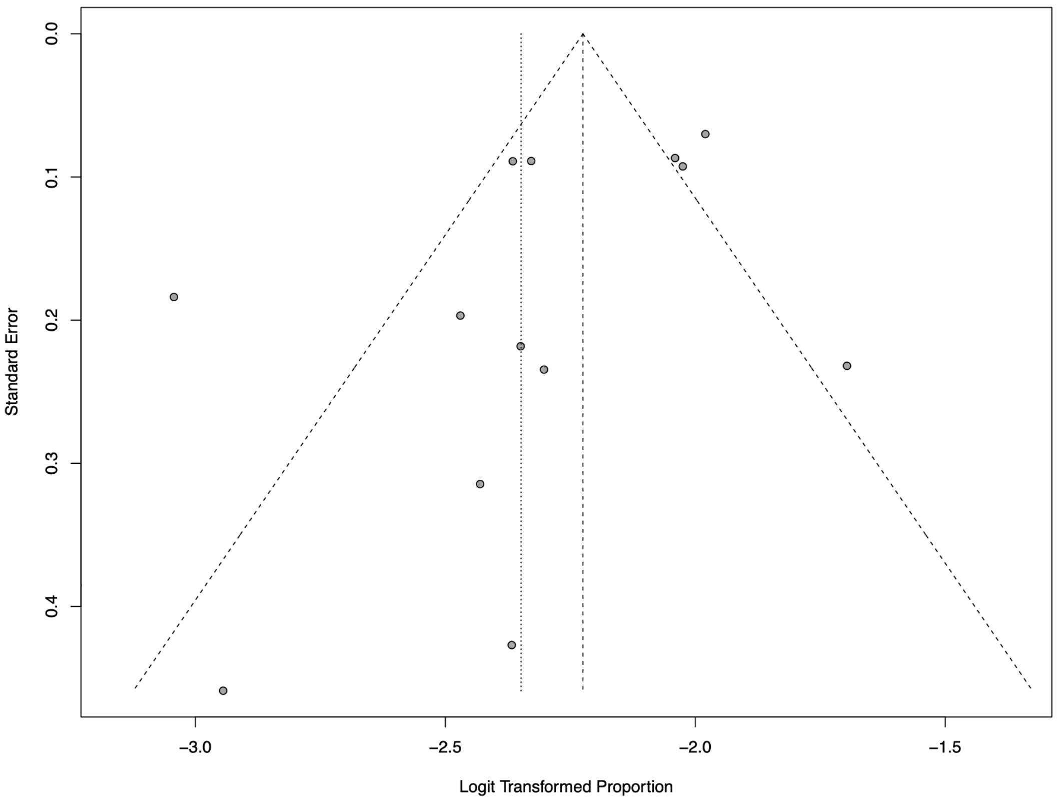

Figure S9 Funnel plots of studies reporting on major adverse cardiac events

Drug-eluting stent. DES: Drug-eluting balloon

DEB studies

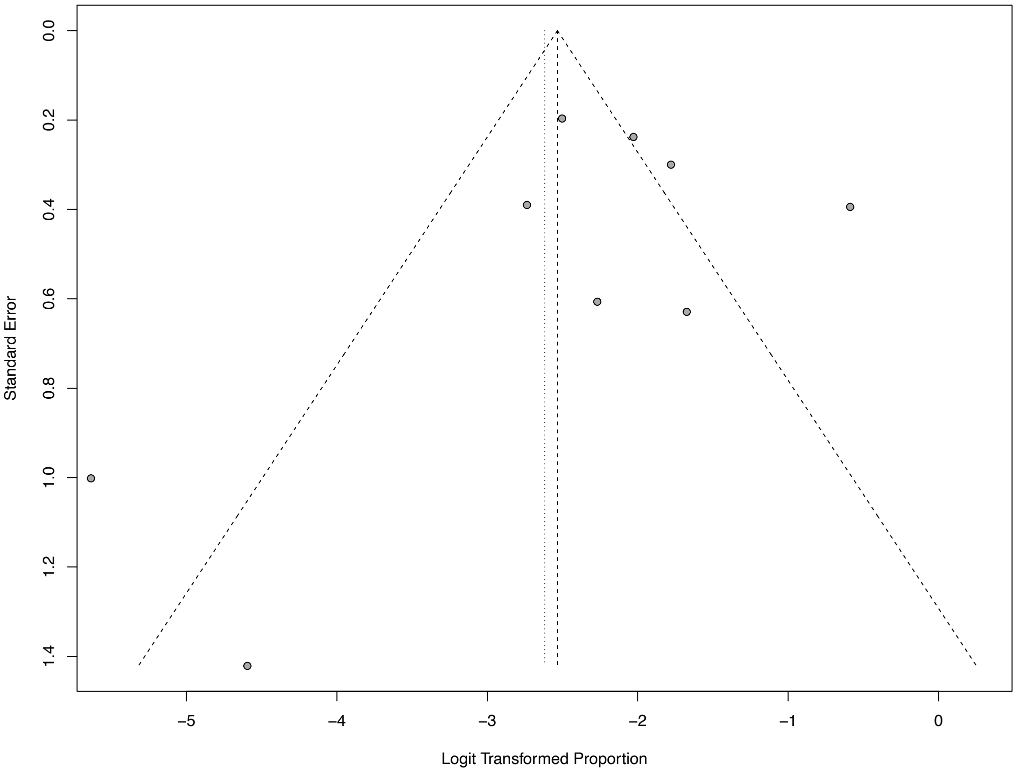

DES studies

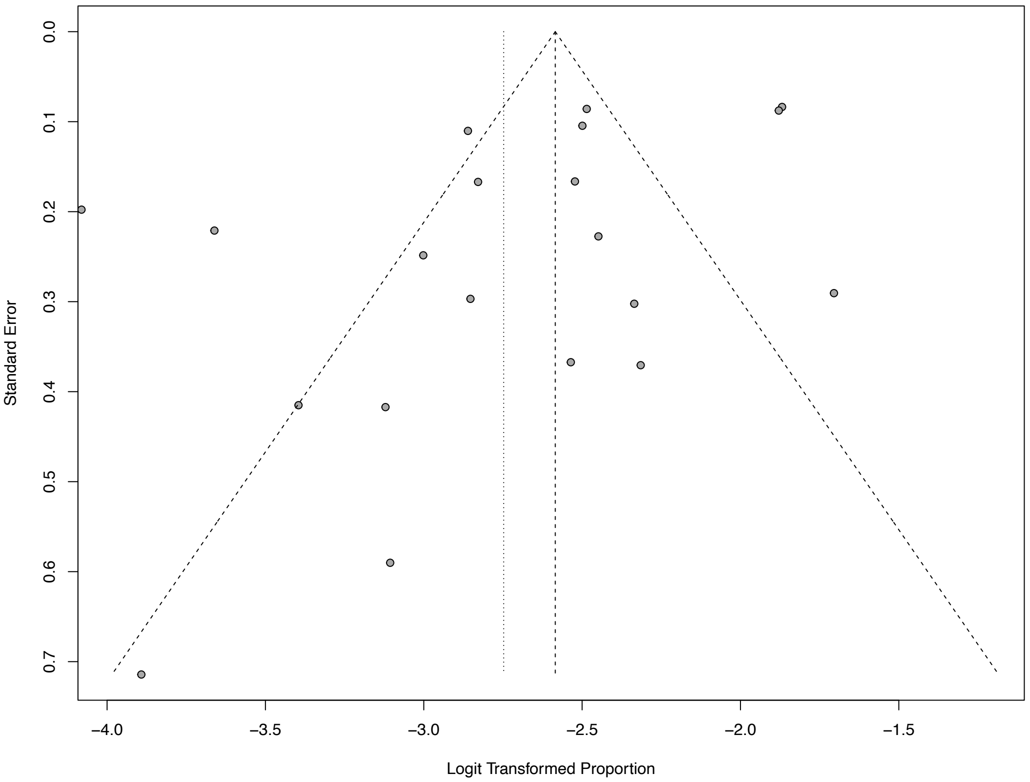

Figure S10 Funnel plots of studies reporting target-vessel revascularizationDEB: Drug-eluting stent. DES: Drug-eluting balloon

DEB studies

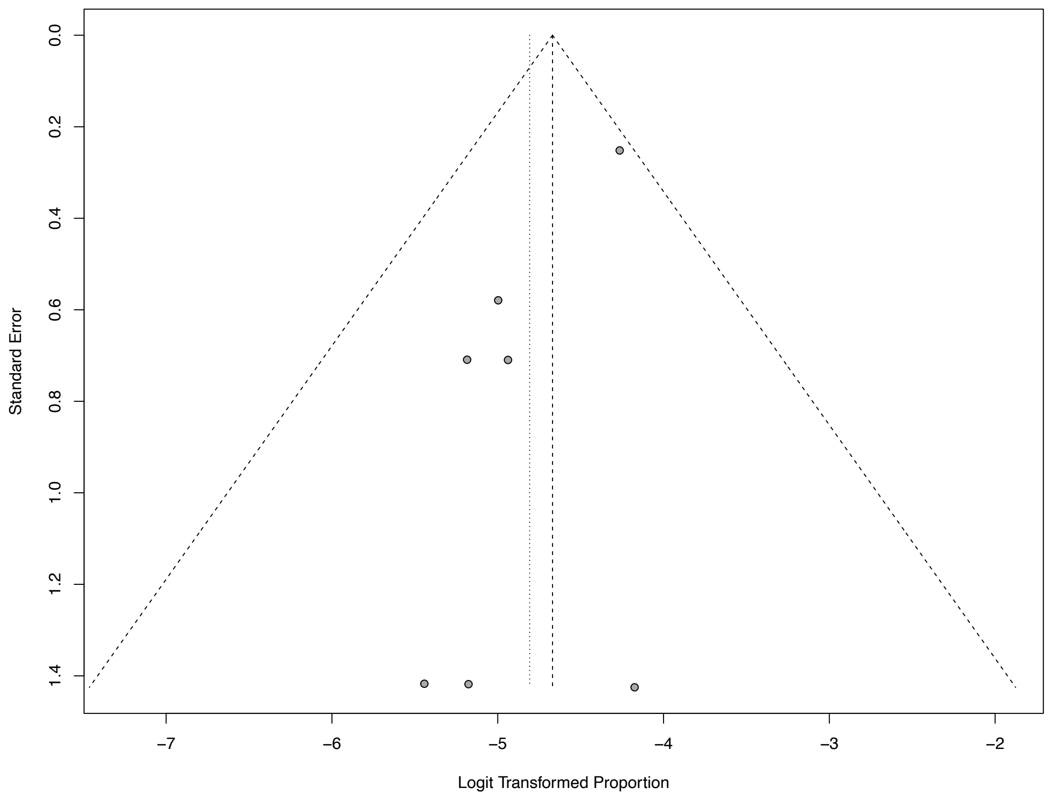

DES studies

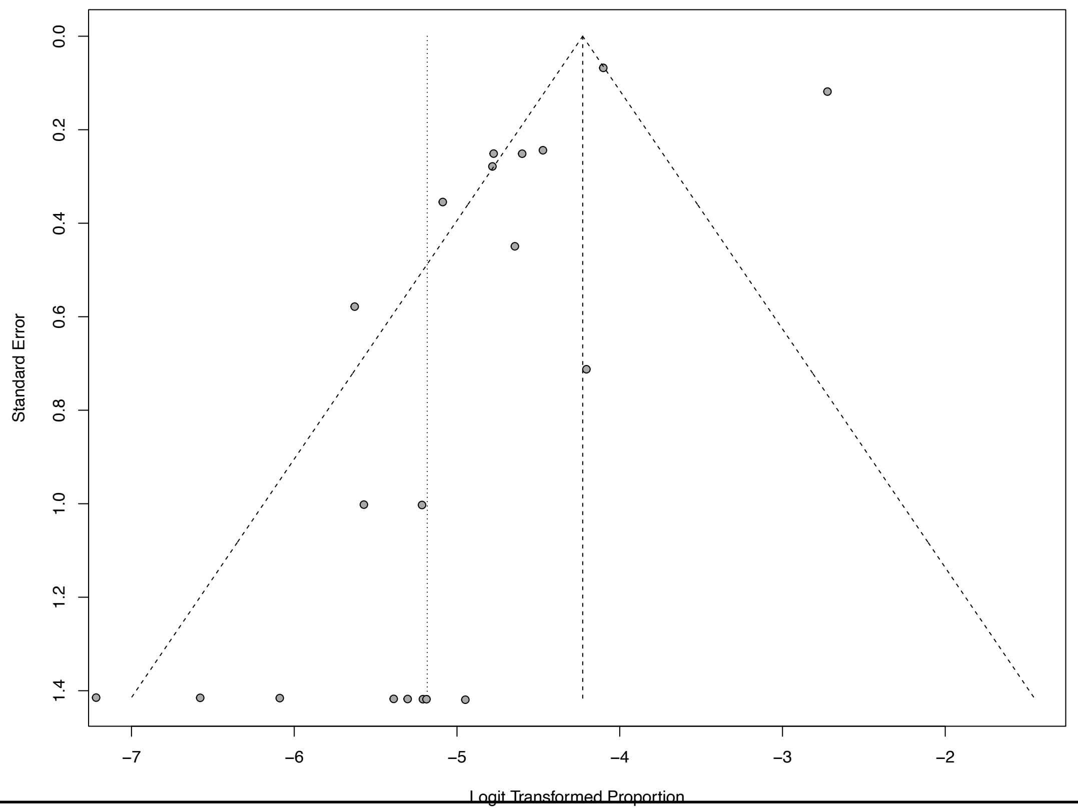

Figure S11 Funnel plots of studies reporting target-lesion thrombosis

DEB: Drug-eluting stent. DES: Drug-eluting balloon

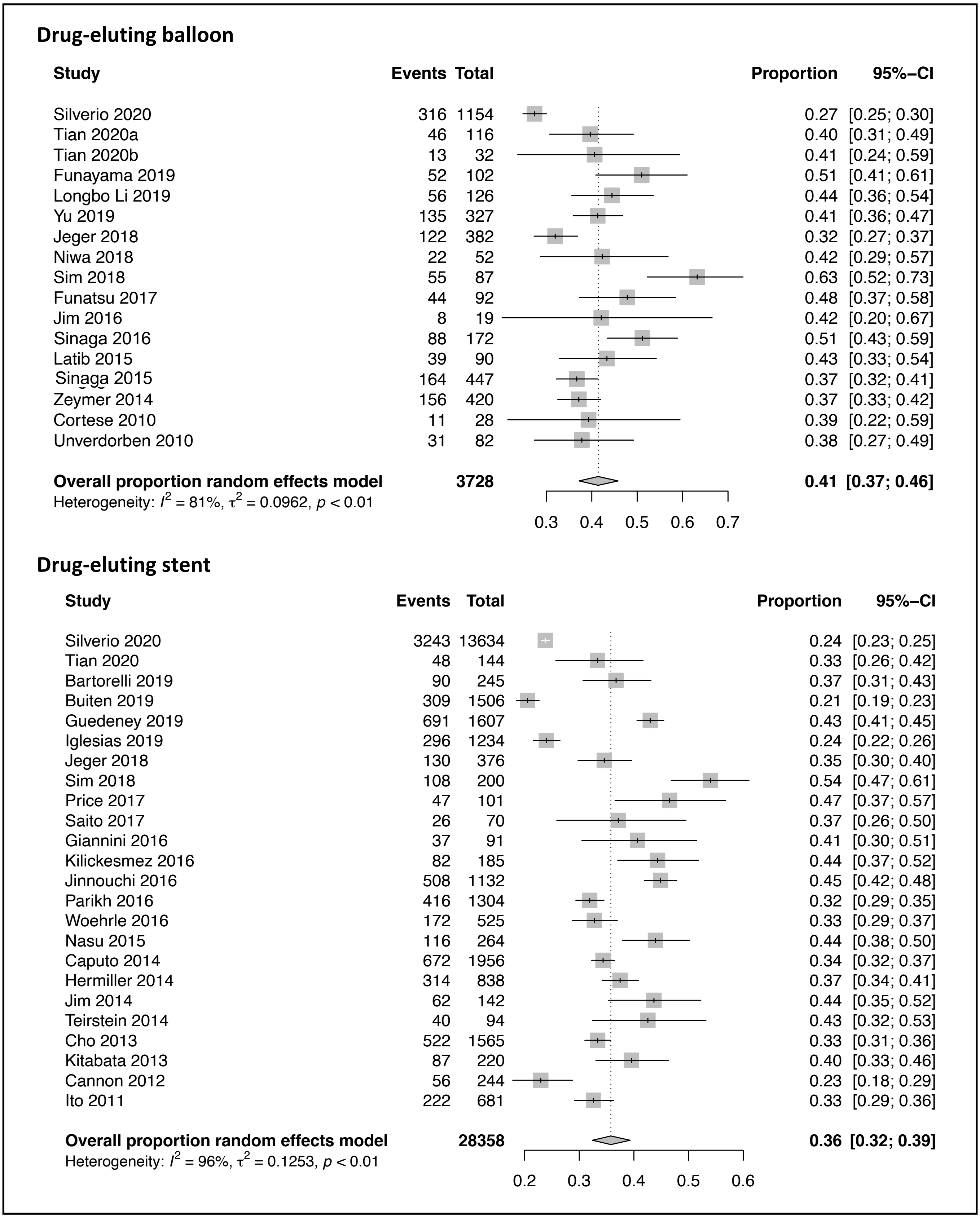

Figure S12 Forest plots of diabetes in patients undergoing DES or DEB for small vessel disease

DEB: Drug-eluting stent. DES: Drug-eluting balloon

Drug-eluting balloon

Study	Events	Total	Proportion	95%–CI
Silverio 2020	143	1154	0.12	[0.11; 0.14]
Tian 2020a	34	116	0.29	[0.21; 0.38]
Tian 2020b	9	32	0.28	[0.14; 0.47]
Funayama 2019	.	102		
Longbo Li 2019	.	126		
Yu 2019	.	327		
Jeger 2018	82	382	0.21	[0.17; 0.26]
Niwa 2018	27	52	0.52	[0.38; 0.66]
Sim 2018	42	87	0.48	[0.37; 0.59]
Funatsu 2017	.	92		
Jim 2016	.	19		
Sinaga 2016	.	172		
Latib 2015	15	90	0.17	[0.10; 0.26]
Sinaga 2015	.	447		
Zeymer 2014	.	420		
Cortese 2010	.	28		
Unverdorben 2010	.	82		
**Overall proportion random effects model**		**3728**	**0.27**	**[0.18; 0.39]**

Heterogeneity: $I^2 = 93\%$, $\tau^2 = 0.4558$, $p < 0.01$

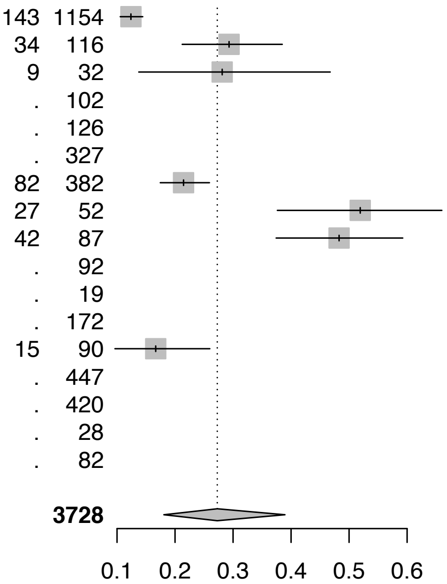

Drug-eluting stent

Study	Events	Total	Proportion	95%–CI
Silverio 2020	2302	13634	0.17	[0.16; 0.18]
Tian 2020	36	144	0.25	[0.18; 0.33]
Bartorelli 2019	124	245	0.51	[0.44; 0.57]
Buiten 2019	390	1506	0.26	[0.24; 0.28]
Guedeney 2019	313	1607	0.19	[0.18; 0.21]
Iglesias 2019	341	1234	0.28	[0.25; 0.30]
Jeger 2018	72	376	0.19	[0.15; 0.23]
Sim 2018	59	200	0.30	[0.23; 0.36]
Price 2017	12	101	0.12	[0.06; 0.20]
Saito 2017	.	70		
Giannini 2016	30	91	0.33	[0.23; 0.44]
Kilickesmez 2016	40	185	0.22	[0.16; 0.28]
Jinnouchi 2016	188	1132	0.17	[0.14; 0.19]
Parikh 2016	273	1304	0.21	[0.19; 0.23]
Woehrle 2016	.	525		
Nasu 2015	33	264	0.12	[0.09; 0.17]
Caputo 2014	409	1956	0.21	[0.19; 0.23]
Hermiller 2014	163	838	0.19	[0.17; 0.22]
Jim 2014	.	142		
Teirstein 2014	16	94	0.17	[0.10; 0.26]
Cho 2013	.	1565		
Kitabata 2013	41	220	0.19	[0.14; 0.24]
Cannon 2012	32	244	0.13	[0.09; 0.18]
Ito 2011	133	681	0.20	[0.17; 0.23]
**Overall proportion random effects model**		**28358**	**0.21**	**[0.18; 0.25]**

Heterogeneity: $I^2 = 96\%$, $\tau^2 = 0.1736$, $p < 0.01$

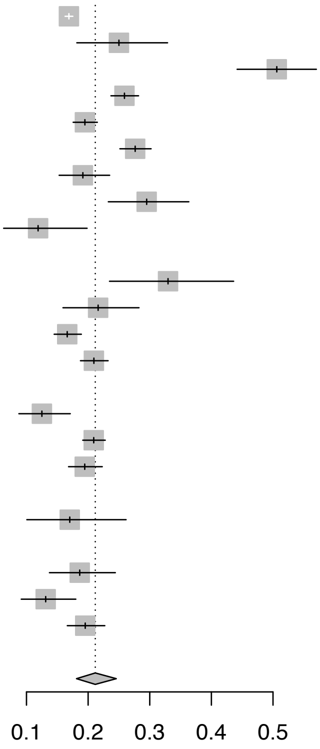

Figure S13 Forest plots of smoking in patients undergoing DES or DEB for small vessel disease

DEB: Drug-eluting stent. DES: Drug-eluting balloon

Drug-eluting balloon

Study	Events	Total	Proportion	95%–CI
Silverio 2020	791	1154	0.69	[0.66; 0.71]
Tian 2020a	78	116	0.67	[0.58; 0.76]
Tian 2020b	26	32	0.81	[0.64; 0.93]
Funayama 2019	81	102	0.79	[0.70; 0.87]
Longbo Li 2019	96	126	0.76	[0.68; 0.83]
Yu 2019	224	327	0.69	[0.63; 0.74]
Jeger 2018	324	382	0.85	[0.81; 0.88]
Niwa 2018	38	52	0.73	[0.59; 0.84]
Sim 2018	69	87	0.79	[0.69; 0.87]
Funatsu 2017	77	92	0.84	[0.75; 0.91]
Jim 2016	13	19	0.68	[0.43; 0.87]
Sinaga 2016	125	172	0.73	[0.65; 0.79]
Latib 2015	72	90	0.80	[0.70; 0.88]
Sinaga 2015	360	447	0.81	[0.77; 0.84]
Zeymer 2014	342	420	0.81	[0.77; 0.85]
Cortese 2010	21	28	0.75	[0.55; 0.89]
Unverdorben 2010	75	82	0.91	[0.83; 0.96]
**Overall proportion random effects model**		**3728**	**0.78**	**[0.74; 0.81]**

Heterogeneity: $I^2 = 79\%$, $\tau^2 = 0.1077$, $p < 0.01$

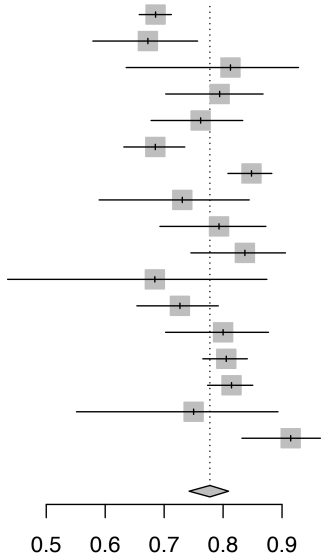

Drug-eluting stent

Study	Events	Total	Proportion	95%–CI
Silverio 2020	8779	13634	0.64	[0.64; 0.65]
Tian 2020	86	144	0.60	[0.51; 0.68]
Bartorelli 2019	189	245	0.77	[0.71; 0.82]
Buiten 2019	729	1506	0.48	[0.46; 0.51]
Guedeney 2019	1335	1607	0.83	[0.81; 0.85]
Iglesias 2019	829	1234	0.67	[0.64; 0.70]
Jeger 2018	332	376	0.88	[0.85; 0.91]
Sim 2018	136	200	0.68	[0.61; 0.74]
Price 2017	83	101	0.82	[0.73; 0.89]
Saito 2017	61	70	0.87	[0.77; 0.94]
Giannini 2016	74	91	0.81	[0.72; 0.89]
Kilickesmez 2016	97	185	0.52	[0.45; 0.60]
Jinnouchi 2016	912	1132	0.81	[0.78; 0.83]
Parikh 2016	1001	1304	0.77	[0.74; 0.79]
Woehrle 2016	375	519	0.72	[0.68; 0.76]
Nasu 2015	209	264	0.79	[0.74; 0.84]
Caputo 2014	1506	1956	0.77	[0.75; 0.79]
Hermiller 2014	719	838	0.86	[0.83; 0.88]
Jim 2014	93	142	0.65	[0.57; 0.73]
Teirstein 2014	75	94	0.80	[0.70; 0.87]
Cho 2013	859	1565	0.55	[0.52; 0.57]
Kitabata 2013	199	220	0.90	[0.86; 0.94]
Cannon 2012	118	244	0.48	[0.42; 0.55]
Ito 2011	542	681	0.80	[0.76; 0.83]
**Overall proportion random effects model**		**28352**	**0.74**	**[0.69; 0.79]**

Heterogeneity: $I^2 = 99\%$, $\tau^2 = 0.3939$, $p < 0.01$

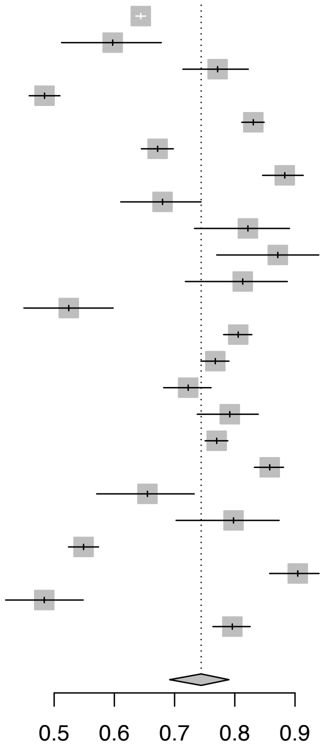

Figure S14 Forest plots of hypertension in patients undergoing DES or DEB for small vessel disease

DEB: Drug-eluting stent. DES: Drug-eluting balloon

Drug-eluting balloon

Study	Events	Total	Proportion	95%-CI
Silverio 2020	680	1154	0.59	[0.56; 0.62]
Tian 2020a	61	116	0.53	[0.43; 0.62]
Tian 2020b	21	32	0.66	[0.47; 0.81]
Funayama 2019	76	102	0.75	[0.65; 0.83]
Longbo Li 2019	91	126	0.72	[0.64; 0.80]
Yu 2019	185	327	0.57	[0.51; 0.62]
Jeger 2018	262	382	0.69	[0.64; 0.73]
Niwa 2018	30	52	0.58	[0.43; 0.71]
Sim 2018	70	87	0.80	[0.71; 0.88]
Funatsu 2017	68	92	0.74	[0.64; 0.83]
Jim 2016	15	19	0.79	[0.54; 0.94]
Sinaga 2016	120	172	0.70	[0.62; 0.77]
Latib 2015	71	90	0.79	[0.69; 0.87]
Sinaga 2015	308	447	0.69	[0.64; 0.73]
Zeymer 2014	292	420	0.70	[0.65; 0.74]
Cortese 2010	17	28	0.61	[0.41; 0.78]
Unverdorben 2010	66	82	0.80	[0.70; 0.88]
**Overall proportion random effects model**		**3728**	**0.69**	**[0.64; 0.73]**

Heterogeneity: $I^2 = 82\%$, $\tau^2 = 0.1090$, $p < 0.01$

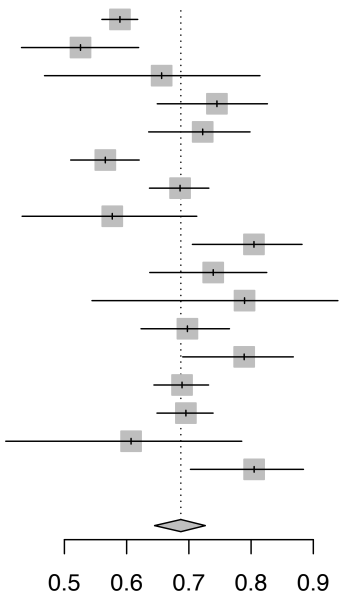

Drug-eluting stent

Study	Events	Total	Proportion	95%-CI
Silverio 2020	6899	13634	0.51	[0.50; 0.51]
Tian 2020	55	144	0.38	[0.30; 0.47]
Bartorelli 2019	142	245	0.58	[0.52; 0.64]
Buiten 2019	605	1506	0.40	[0.38; 0.43]
Guedeney 2019	1265	1607	0.79	[0.77; 0.81]
Iglesias 2019	824	1234	0.67	[0.64; 0.69]
Jeger 2018	259	376	0.69	[0.64; 0.74]
Sim 2018	153	200	0.76	[0.70; 0.82]
Price 2017	95	101	0.94	[0.88; 0.98]
Saito 2017	61	70	0.87	[0.77; 0.94]
Giannini 2016	71	91	0.78	[0.68; 0.86]
Kilickesmez 2016	69	185	0.37	[0.30; 0.45]
Jinnouchi 2016	805	1132	0.71	[0.68; 0.74]
Parikh 2016	941	1304	0.72	[0.70; 0.75]
Woehrle 2016	360	515	0.70	[0.66; 0.74]
Nasu 2015	178	264	0.67	[0.61; 0.73]
Caputo 2014	1497	1956	0.77	[0.75; 0.78]
Hermiller 2014	690	838	0.82	[0.80; 0.85]
Jim 2014	81	142	0.57	[0.48; 0.65]
Teirstein 2014	77	94	0.82	[0.73; 0.89]
Cho 2013	616	1565	0.39	[0.37; 0.42]
Kitabata 2013	196	220	0.89	[0.84; 0.93]
Cannon 2012	122	244	0.50	[0.44; 0.56]
Ito 2011	545	681	0.80	[0.77; 0.83]
**Overall proportion random effects model**		**28348**	**0.69**	**[0.62; 0.76]**

Heterogeneity: $I^2 = 99\%$, $\tau^2 = 0.6472$, $p = 0$

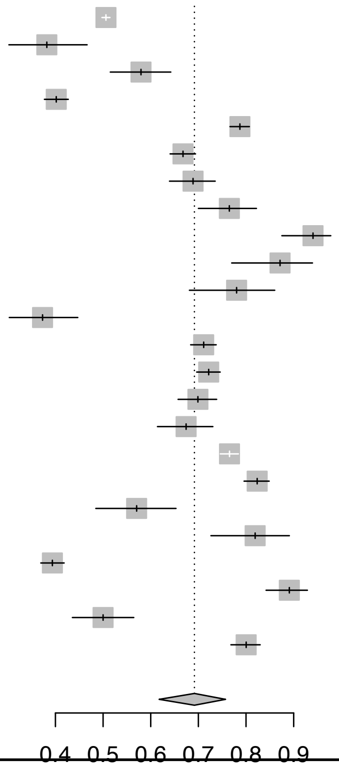

Figure S15 Forest plots of dyslipidemia in patients undergoing DES or DEB for small vessel disease

DEB: Drug-eluting stent. DES: Drug-eluting balloon

Drug-eluting balloon

Study	Events	Total	Proportion	95%-CI
Silverio 2020	365	1154	0.32	[0.29; 0.34]
Tian 2020a	26	116	0.22	[0.15; 0.31]
Tian 2020b	8	32	0.25	[0.11; 0.43]
Funayama 2019	31	102	0.30	[0.22; 0.40]
Longbo Li 2019	25	126	0.20	[0.13; 0.28]
Yu 2019	.	327		
Jeger 2018	160	382	0.42	[0.37; 0.47]
Niwa 2018	2	52	0.04	[0.00; 0.13]
Sim 2018	.	87		
Funatsu 2017	.	92		
Jim 2016	3	19	0.16	[0.03; 0.40]
Sinaga 2016	.	172		
Latib 2015	46	90	0.51	[0.40; 0.62]
Sinaga 2015	.	447		
Zeymer 2014	.	420		
Cortese 2010	5	28	0.18	[0.06; 0.37]
Unverdorben 2010	.	82		
**Overall proportion random effects model**		**3728**	**0.25**	**[0.17; 0.35]**

Heterogeneity: $I^2 = 93\%$, $\tau^2 = 0.4525$, $p < 0.01$

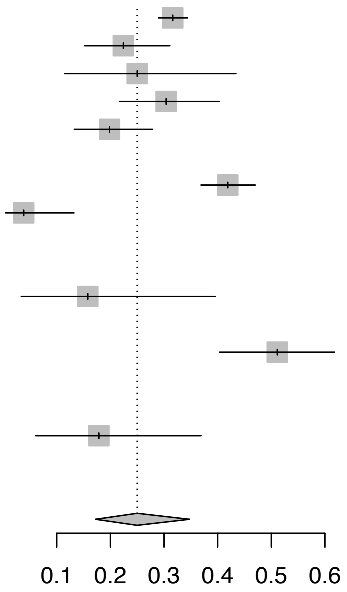

Drug-eluting stent

Study	Events	Total	Proportion	95%-CI
Silverio 2020	3386	13634	0.25	[0.24; 0.26]
Tian 2020	28	144	0.19	[0.13; 0.27]
Bartorelli 2019	64	245	0.26	[0.21; 0.32]
Buiten 2019	291	1506	0.19	[0.17; 0.21]
Guedeney 2019	743	1607	0.46	[0.44; 0.49]
Iglesias 2019	251	1234	0.20	[0.18; 0.23]
Jeger 2018	133	376	0.35	[0.31; 0.40]
Sim 2018	.	200		
Price 2017	35	101	0.35	[0.25; 0.45]
Saito 2017	19	70	0.27	[0.17; 0.39]
Giannini 2016	53	91	0.58	[0.47; 0.68]
Kilickesmez 2016	43	185	0.23	[0.17; 0.30]
Jinnouchi 2016	250	1132	0.22	[0.20; 0.25]
Parikh 2016	352	1304	0.27	[0.25; 0.29]
Woehrle 2016	169	525	0.32	[0.28; 0.36]
Nasu 2015	63	264	0.24	[0.19; 0.29]
Caputo 2014	529	1956	0.27	[0.25; 0.29]
Hermiller 2014	251	838	0.30	[0.27; 0.33]
Jim 2014	28	142	0.20	[0.14; 0.27]
Teirstein 2014	28	94	0.30	[0.21; 0.40]
Cho 2013	.	1565		
Kitabata 2013	63	220	0.29	[0.23; 0.35]
Cannon 2012	39	244	0.16	[0.12; 0.21]
Ito 2011	159	681	0.23	[0.20; 0.27]
**Overall proportion random effects model**		**28358**	**0.27**	**[0.24; 0.31]**

Heterogeneity: $I^2 = 96\%$, $\tau^2 = 0.1580$, $p < 0.01$

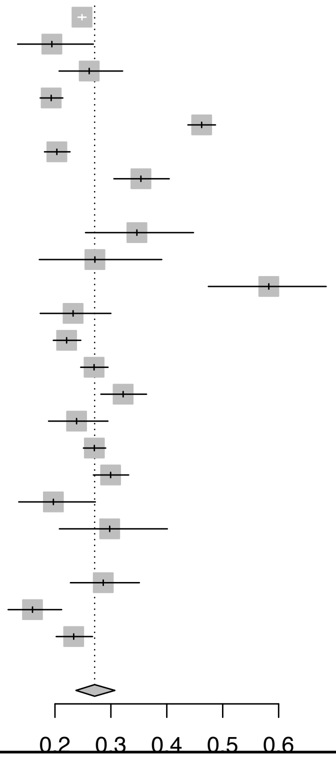

Figure S16 Forest plots of previous myocardial infarction in patients undergoing DES or DEB for small vessel disease

DEB: Drug-eluting stent. DES: Drug-eluting balloon

Drug-eluting balloon

Study	Events	Total	Proportion	95%–CI
Silverio 2020	394	1154	0.34	[0.31; 0.37]
Tian 2020a	45	116	0.39	[0.30; 0.48]
Tian 2020b	6	32	0.19	[0.07; 0.36]
Funayama 2019	62	102	0.61	[0.51; 0.70]
Longbo Li 2019	38	126	0.30	[0.22; 0.39]
Yu 2019	24	327	0.07	[0.05; 0.11]
Jeger 2018	235	382	0.62	[0.56; 0.66]
Niwa 2018	27	52	0.52	[0.38; 0.66]
Sim 2018	32	87	0.37	[0.27; 0.48]
Funatsu 2017	.	92		
Jim 2016	4	19	0.21	[0.06; 0.46]
Sinaga 2016	.	172		
Latib 2015	52	90	0.58	[0.47; 0.68]
Sinaga 2015	238	447	0.53	[0.48; 0.58]
Zeymer 2014	225	420	0.54	[0.49; 0.58]
Cortese 2010	3	28	0.11	[0.02; 0.28]
Unverdorben 2010	.	82		
**Overall proportion random effects model**		**3728**	**0.37**	**[0.26; 0.48]**

Heterogeneity: $I^2 = 97\%$, $\tau^2 = 0.7654$, $p < 0.01$

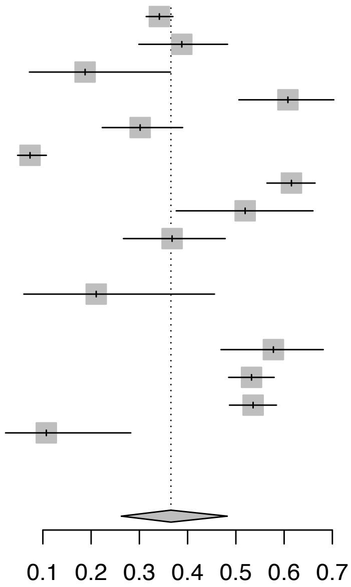

Drug-eluting stent

Study	Events	Total	Proportion	95%–CI
Silverio 2020	3266	13634	0.24	[0.23; 0.25]
Tian 2020	38	144	0.26	[0.19; 0.34]
Bartorelli 2019	97	245	0.40	[0.33; 0.46]
Buiten 2019	282	1506	0.19	[0.17; 0.21]
Guedeney 2019	779	1607	0.48	[0.46; 0.51]
Iglesias 2019	365	1234	0.30	[0.27; 0.32]
Jeger 2018	241	376	0.64	[0.59; 0.69]
Sim 2018	48	200	0.24	[0.18; 0.31]
Price 2017	60	101	0.59	[0.49; 0.69]
Saito 2017	37	70	0.53	[0.41; 0.65]
Giannini 2016	52	91	0.57	[0.46; 0.67]
Kilickesmez 2016	40	185	0.22	[0.16; 0.28]
Jinnouchi 2016	503	1132	0.44	[0.42; 0.47]
Parikh 2016	379	1304	0.29	[0.27; 0.32]
Woehrle 2016	214	525	0.41	[0.37; 0.45]
Nasu 2015	105	264	0.40	[0.34; 0.46]
Caputo 2014	648	1956	0.33	[0.31; 0.35]
Hermiller 2014	360	838	0.43	[0.40; 0.46]
Jim 2014	.	142		
Teirstein 2014	.	94		
Cho 2013	.	1565		
Kitabata 2013	81	220	0.37	[0.30; 0.44]
Cannon 2012	.	244		
Ito 2011	.	681		
**Overall proportion random effects model**		**28358**	**0.38**	**[0.32; 0.44]**

Heterogeneity: $I^2 = 98\%$, $\tau^2 = 0.3083$, $p < 0.01$

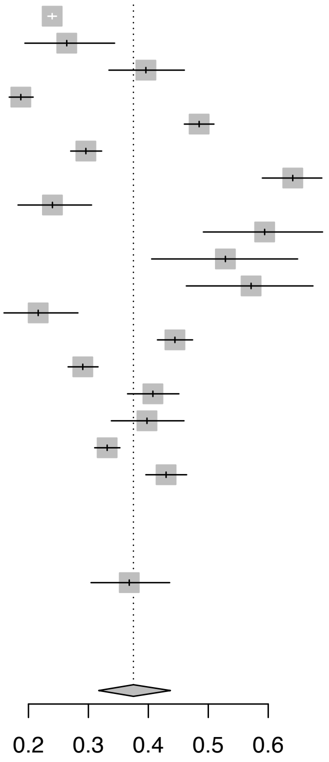

Figure S17 Forest plots of previous PCI in patients undergoing DES or DEB for small vessel disease

DEB: Drug-eluting stent. DES: Drug-eluting balloon. PCI: Percutaneous coronary intervention

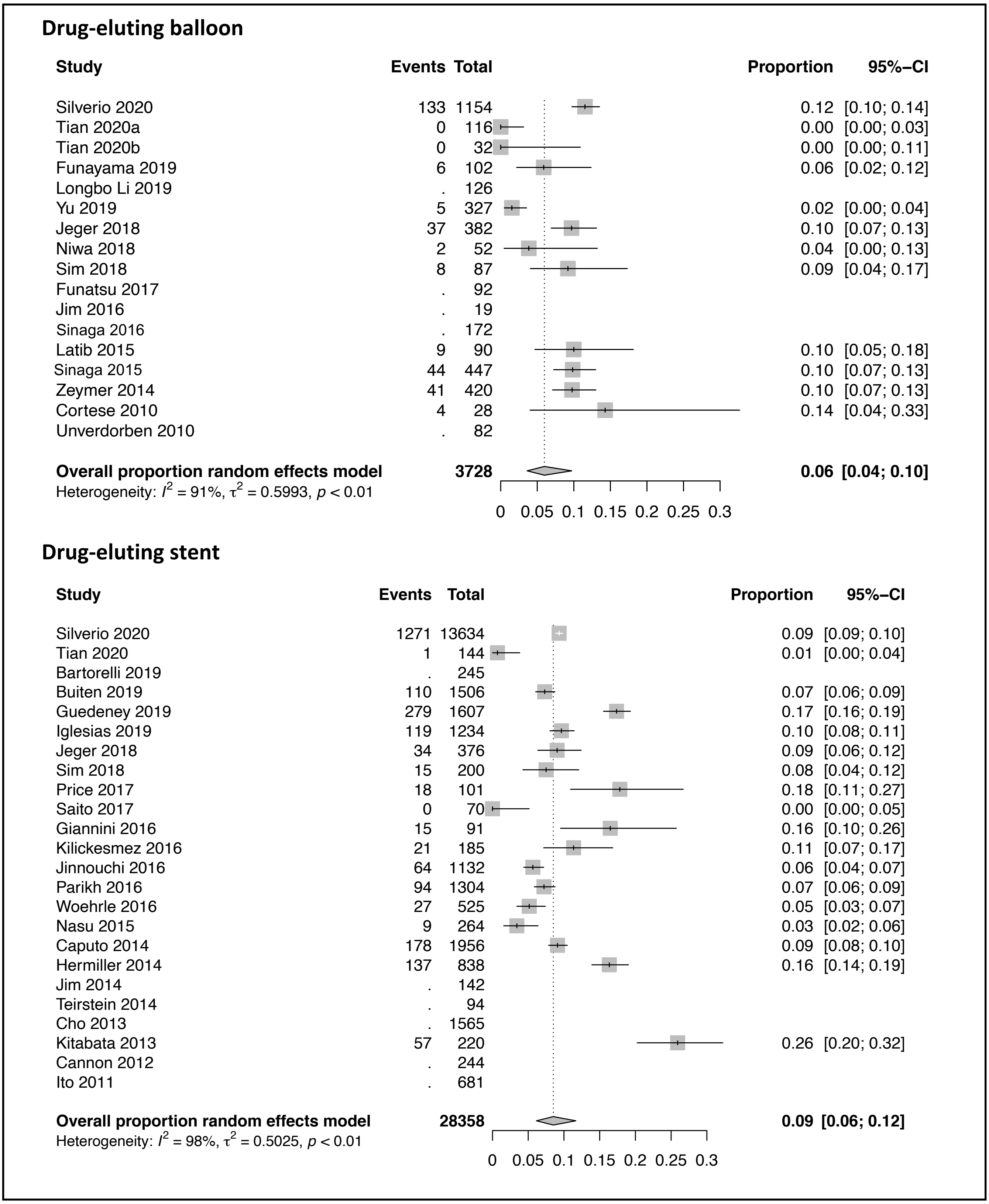

Figure S18 Forest plots of CABG in patients undergoing DES or DEB for small vessel disease

DEB: Drug-eluting stent. DES: Drug-eluting balloon. CABG: Coronary artery bypass graft

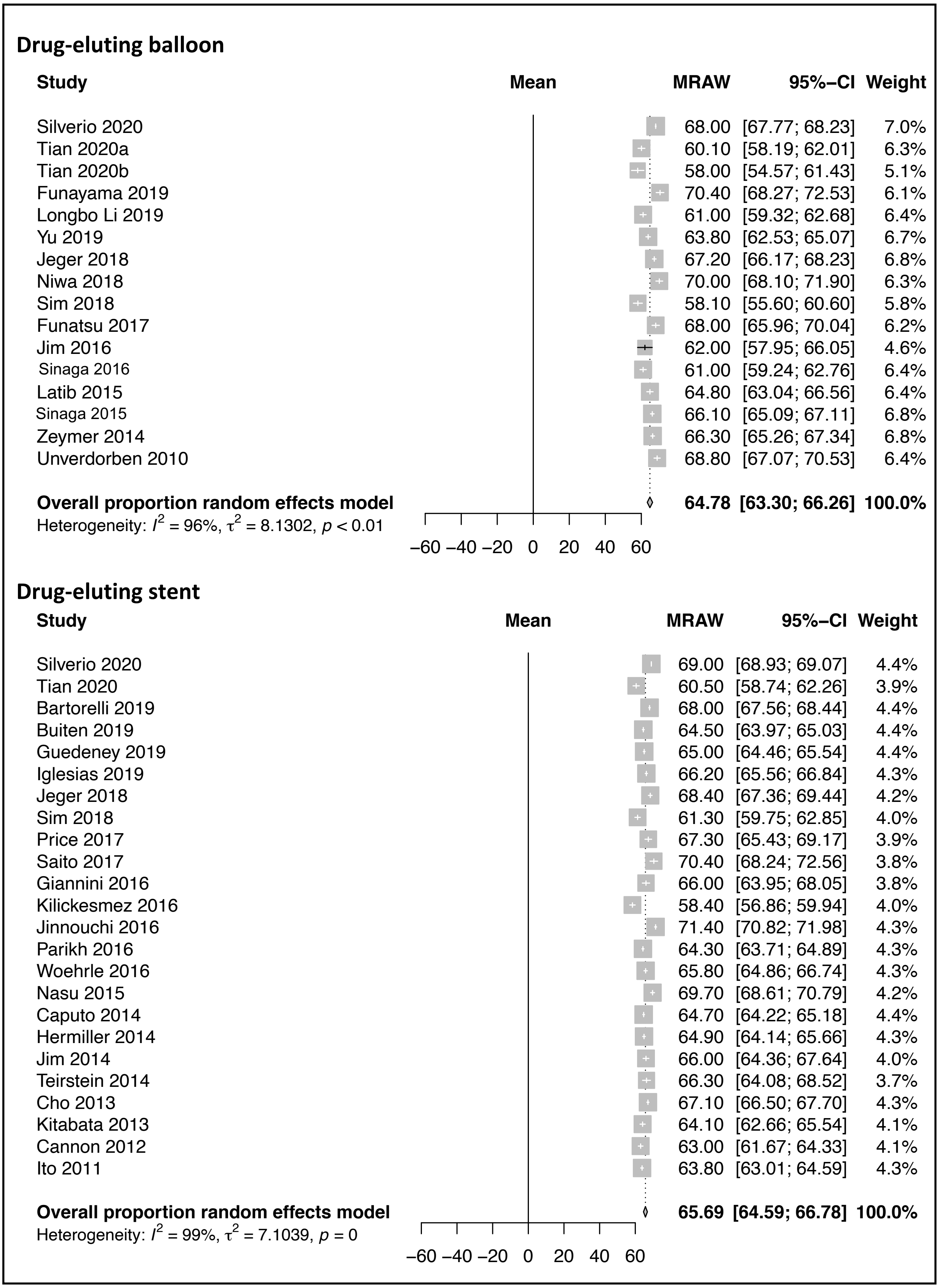

Figure S19 Forest plots of age in patients undergoing DES or DEB for small vessel disease

DEB: Drug-eluting stent. DES: Drug-eluting balloon

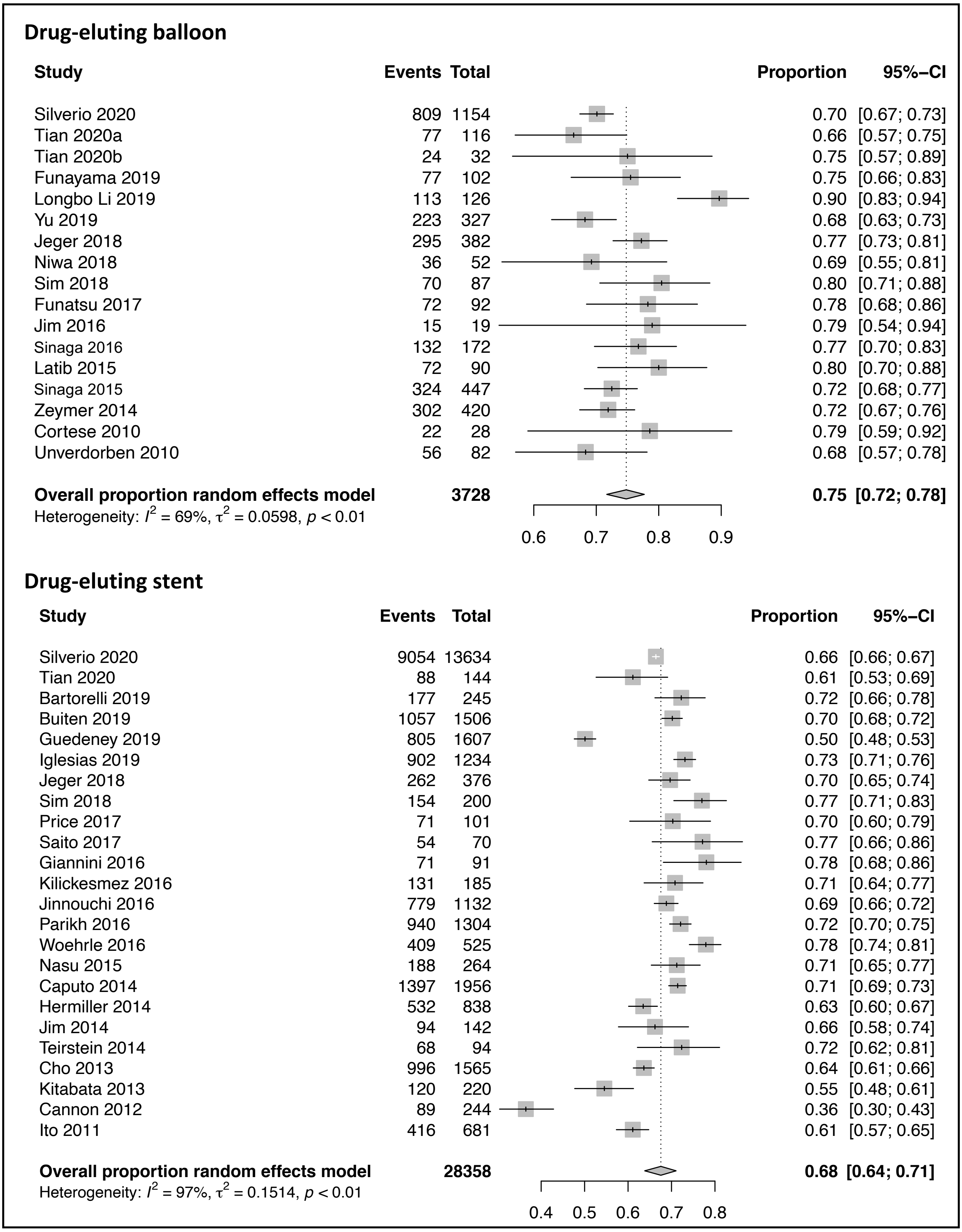

Figure S20 Forest plots of male gender in patients undergoing DES or DEB for small vessel disease

DEB: Drug-eluting stent. DES: Drug-eluting balloon

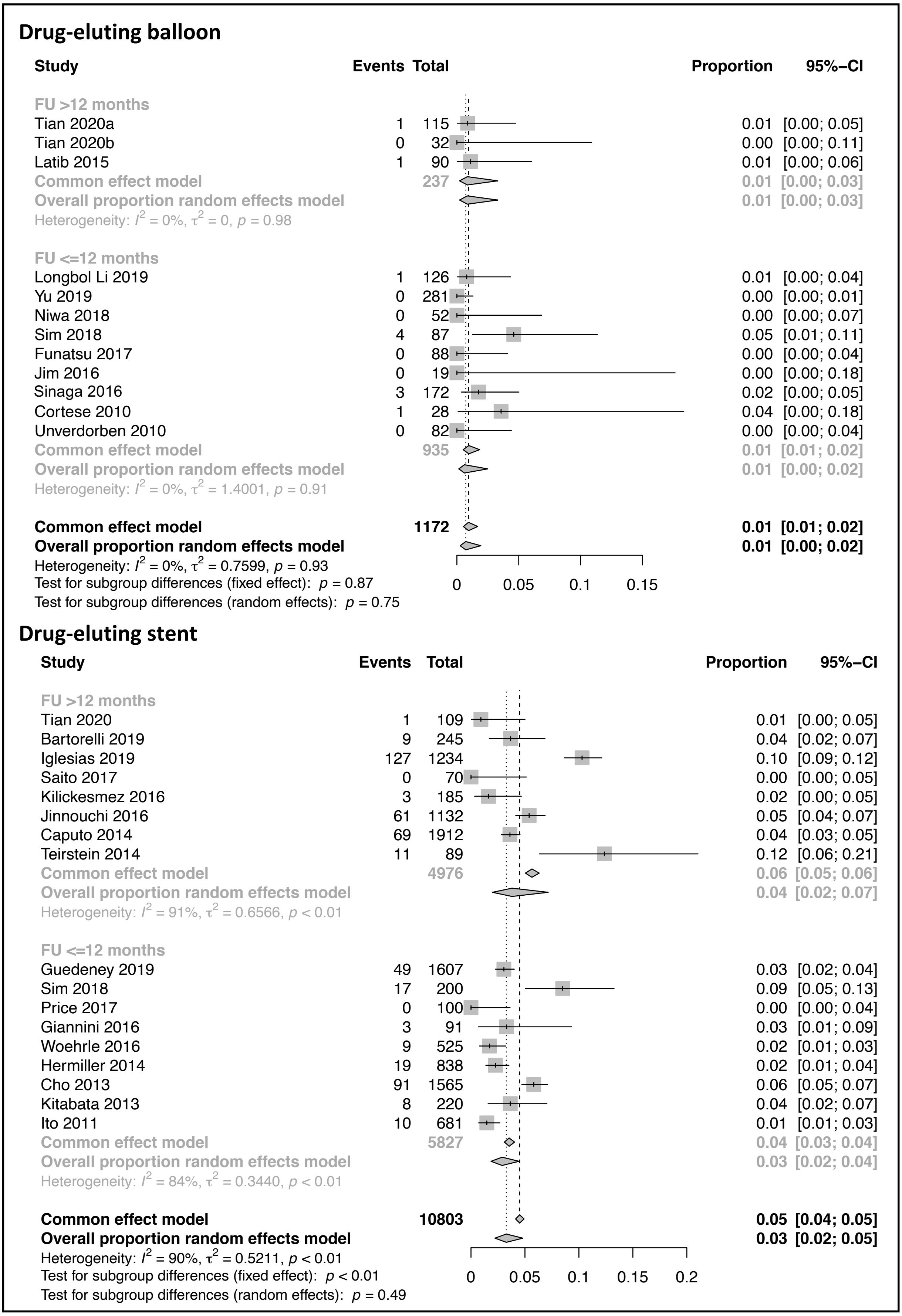

Figure S21 Forest plots of all-cause mortality in patients undergoing DES or DEB for small vessel disease after exclusion of Silverio et al.

DEB: Drug-eluting stent. DES: Drug-eluting balloon

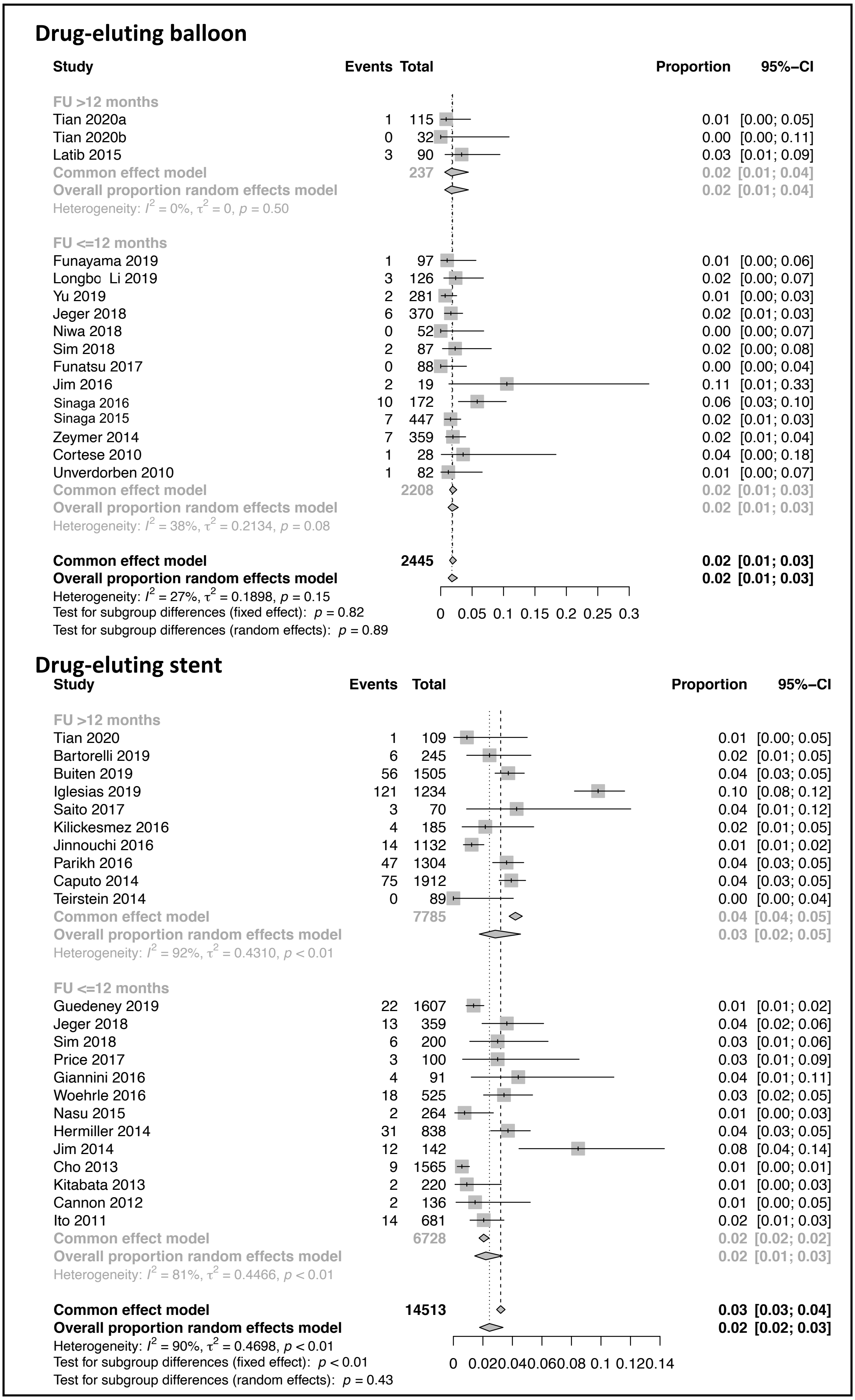

Figure S22 Forest plots of myocardial infarction in patients undergoing DES or DEB for small vessel disease after exclusion of Silverio et al.

DEB: Drug-eluting stent. DES: Drug-eluting balloon

Drug-eluting balloon

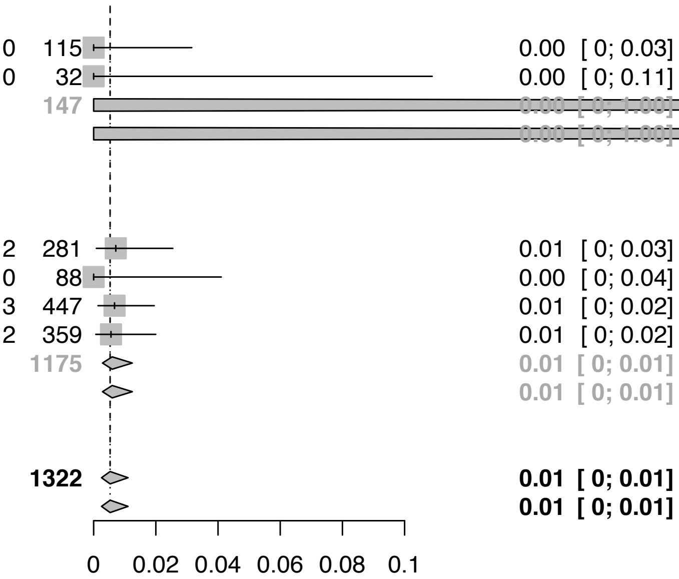

Study	Events	Total	Proportion	95%-CI
**FU >12 months**				
Tian 2020a	0	115	0.00	[0; 0.03]
Tian 2020b	0	32	0.00	[0; 0.11]
**Common effect model**		147	0.00	[0; 1.00]
**Overall proportion random effects model**			0.00	[0; 1.00]
Heterogeneity: $I^2 = 0\%$, $\tau^2 = 0$, $p = 1.00$				
**FU <=12 months**				
Yu 2019	2	281	0.01	[0; 0.03]
Funatsu 2017	0	88	0.00	[0; 0.04]
Sinaga 2015	3	447	0.01	[0; 0.02]
Zeymer 2014	2	359	0.01	[0; 0.02]
**Common effect model**		1175	0.01	[0; 0.01]
**Overall proportion random effects model**			0.01	[0; 0.01]
Heterogeneity: $I^2 = 0\%$, $\tau^2 = 0$, $p = 1.00$				
**Common effect model**		**1322**	**0.01**	**[0; 0.01]**
**Overall proportion random effects model**			**0.01**	**[0; 0.01]**

Heterogeneity: $I^2 = 0\%$, $\tau^2 = 0$, $p = 1.00$
Test for subgroup differences (fixed effect): $p = 1.00$
Test for subgroup differences (random effects): $p = 1.00$

Drug-eluting balloon

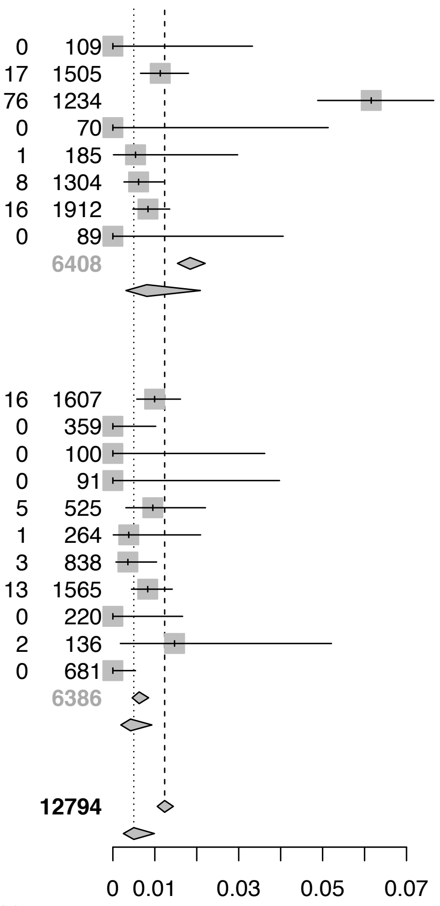

Study	Events	Total	Proportion	95%-CI
**FU >12 months**				
Tian 2020	0	109	0.00	[0.00; 0.03]
Buiten 2019	17	1505	0.01	[0.01; 0.02]
Iglesias 2019	76	1234	0.06	[0.05; 0.08]
Saito 2017	0	70	0.00	[0.00; 0.05]
Kilickesmez 2016	1	185	0.01	[0.00; 0.03]
Parikh 2016	8	1304	0.01	[0.00; 0.01]
Caputo 2014	16	1912	0.01	[0.00; 0.01]
Teirstein 2014	0	89	0.00	[0.00; 0.04]
**Common effect model**		6408	0.02	[0.02; 0.02]
**Overall proportion random effects model**			0.01	[0.00; 0.02]
Heterogeneity: $I^2 = 93\%$, $\tau^2 = 1.0310$, $p < 0.01$				
**FU <=12 months**				
Guedeney 2019	16	1607	0.01	[0.01; 0.02]
Jeger 2018	0	359	0.00	[0.00; 0.01]
Price 2017	0	100	0.00	[0.00; 0.04]
Giannini 2016	0	91	0.00	[0.00; 0.04]
Woehrle 2016	5	525	0.01	[0.00; 0.02]
Nasu 2015	1	264	0.00	[0.00; 0.02]
Hermiller 2014	3	838	0.00	[0.00; 0.01]
Cho 2013	13	1565	0.01	[0.00; 0.01]
Kitabata 2013	0	220	0.00	[0.00; 0.02]
Cannon 2012	2	136	0.01	[0.00; 0.05]
Ito 2011	0	681	0.00	[0.00; 0.01]
**Common effect model**		6386	0.01	[0.00; 0.01]
**Overall proportion random effects model**			0.00	[0.00; 0.01]
Heterogeneity: $I^2 = 0\%$, $\tau^2 = 0.4773$, $p = 0.95$				
**Common effect model**		**12794**	**0.01**	**[0.01; 0.01]**
**Overall proportion random effects model**			**0.00**	**[0.00; 0.01]**

Heterogeneity: $I^2 = 89\%$, $\tau^2 = 1.0934$, $p < 0.01$
Test for subgroup differences (fixed effect): $p < 0.01$
Test for subgroup differences (random effects): $p = 0.30$

Figure S23 Forest plots of target lesion revascularisation in patients undergoing DES or DEB for small vessel disease after exclusion of Silverio et al.

DEB: Drug-eluting stent. DES: Drug-eluting balloon
